# Driving climate resilience: citizen attitudes toward agroforestry and their policy implications in the UK

**DOI:** 10.1007/s13593-026-01130-w

**Published:** 2026-07-13

**Authors:** Shan Jin, Rao Fu, Kyriaki Remoundou, Novieta H. Sari, Meng Yue, Yit Arn Teh, Felix Eigenbrod, Francisco J. Areal, Lynn J. Frewer

**Affiliations:** 1 Royal Agricultural University, Cirencester, GL7 6JS UK; 2 School of Natural and Environmental Sciences, Newcastle University, Newcastle upon Tyne, NE1 7RU UK; 3 Aberystwyth Business School, Penglais Campus, Aberystwyth University, Aberystwyth, SY23 3DY UK; 4 School of Economics and Trade, Hubei University of Economics, Wuhan, 430205 PR China; 5 School of Geography and Environmental Science, University of Southampton, Southampton, SO17 1BJ UK; 6 Newcastle Business School, Northumbria University, Newcastle upon Tyne, NE1 8ST UK

**Keywords:** Agroforestry, Citizen attitude, Climate resilience, Benefit perception, Risk perception, Rural landscape, Place attachment, Latent class analysis, Structural equation modeling

## Abstract

Agroforestry provides essential ecosystem services while enhancing climate resilience and biodiversity in rural landscapes. Despite a growing body of research on farmers’ adoption of agroforestry, much less is known about citizens’ attitudes and priorities. Citizen disapproval of agroforestry practices or policies may undermine the legitimacy of these initiatives and impede their long-term implementation. This risk is particularly relevant in countries where agroforestry is being promoted as a national policy priority, such as the UK. We surveyed 1509 UK citizens to examine their attitudes toward agroforestry, explore factors influencing these attitudes, and study how these factors operate across distinct segments of the sample. Overall, participants expressed neutral to positive attitudes toward agroforestry. Benefits associated with climate resilience (wildlife habitat creation, greenhouse gas capture, and flood control) were rated most highly. Negative socio-economic consequences (delayed benefit realization, higher input requirements, and potential stakeholder conflicts) were identified as representing the greatest risks. Structural equation modeling validated a novel hybrid model explaining participants’ attitudes toward agroforestry, highlighting three primary drivers: evoked affect, perceived benefits, and the perceived importance of environmental conservation in farming. Latent class analysis identified three distinct citizen groups. The negative impact of perceived risk associated with agroforestry on attitudes was greatest among *cautious conservation-oriented citizens*. The positive impact of perceived threat to the rural environment exceeded the influence of perceived importance of environmental conservation in farming among *citizens sensitive to threats to the rural environment*. Among *countryside-engaged eco-productive citizens*, perceived importance of both environmental conservation and food productivity as well as attachment to the countryside emerged as strong positive predictors of acceptance. Our results provide evidence for adopting a holistic approach that accounts for diverse citizen preferences when promoting agroforestry across UK regions, and highlight the importance of addressing multiple ecosystem services at the landscape level in agroforestry policies.

**Supplementary Information:**

The online version contains supplementary material available at 10.1007/s13593-026-01130-w.

## Introduction

Agroforestry, whereby trees and shrubs are integrated into agricultural systems, can deliver a range of ecosystem services (ES) (FAO 2025). Rather than viewing environmental sustainability and agricultural productivity as competing priorities, agroforestry seeks to reconcile them: practices such as carbon sequestration and the enhancement of wildlife habitats underpin soil health and food security by fostering nutrient cycling and essential ES, thereby strengthening farming systems against the increasing challenges posed by climate variability (Willmott et al. 2023; Rüegg et al. 2025). Agroforestry, particularly when integrated into agroecological and regenerative practices, enables a long-term transition toward a sociobiodiverse economy, extending far beyond the mere technical integration of trees and their functional benefits. This sociobiodiverse model fundamentally challenges the logic of the industrial plantation economy, which relies on homogenizing standards and high inputs, by introducing a multispecies logic centered on social and ecological diversity and producer autonomy (van der Ploeg 2021; Ollinaho and Kröger 2023). Although agroforestry has been internationally promoted, it may also introduce risks and uncertainties regarding agricultural production, rural landscapes, and farm productivity and income. Changes in land use can impact traditional farming practices, affecting yields and market dynamics (Castle et al. 2021). The cultivation of exotic tree species may provoke significant concerns regarding negative impacts on native wildlife (Drew-Smythe et al. 2023). Over the long term, these tree species, often used in areas that cannot absorb their impacts, may contribute to the destruction of primary forests by reinforcing the dominance of the industrial plantation economy (Ollinaho and Kröger 2021). Agroforestry also presents a unique aesthetic paradox: while researchers often associate increased tree cover with higher landscape quality, piecemeal transformations that clash with traditional rural imagery can diminish perceived value, potentially triggering resistance from local communities and visitors from urban areas (Nassauer 1995; Tempesta 2010; Fagerholm et al. 2016). Furthermore, agroforestry requires a long-term investment as well as knowledge and skills pertaining to planting and managing trees on farms, which may act as barriers to agroforestry adoption (Abdul-Salam et al. 2022; Tranchina et al. 2024).

Various socio-economic and ecological issues related to agroforestry have been identified, which influence farmers’ decisions regarding adoption (e.g., see Jha et al. 2021; Leduc and Hansson 2024; Neupane et al. 2002; Tranchina et al. 2024). However, there is limited research examining citizens’ perspectives and preferences regarding agroforestry practices, including the potential socio-economic and ecological consequences, both positive and negative (Gao et al. 2014; Islam et al. 2015; Gaspar et al. 2016; Otter and Langenberg 2020; Mayele and Bongo 2022; Bamwesigye et al. 2024). Eliciting public preferences and accounting for these preferences in policy design can lead to better policy decisions that reflect people’s values and reduce the risk of societal conflict over future land-use changes (Otter and Langenberg 2020). Notably, regionally differentiated societal conflict may emerge since people’s preferences associated with farming and rural development vary across socio-economic, cultural, and ecological contexts (Li et al. 2021; Areal et al. 2026). Aligning public values with farmer interests could not only provide the social license necessary for the scaling-up of agroforestry but also foster a strategic alliance with farmers seeking to adopt these practices (Meesters et al. 2021). While not sufficient on its own, this alignment could support the political-economic contestation needed to move beyond capital-intensive, single-species paradigms while potentially fostering autonomous, site-specific land use that resists the homogenizing pressures of the industrial plantation economy (Williams et al. 2023; Maryono et al. 2024).

**Fig. 1:**
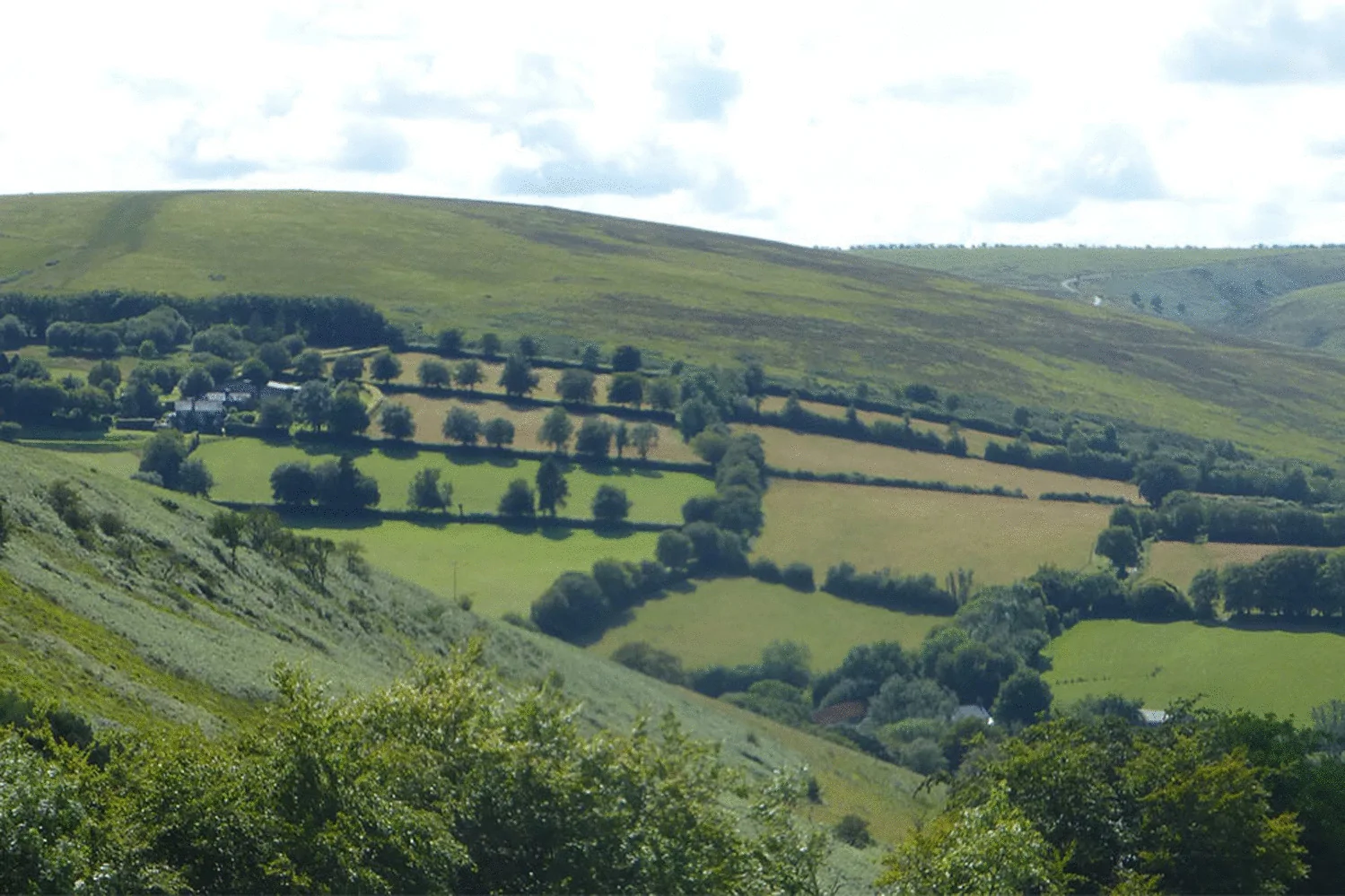
Hedgerows, farm woodlands or forests along rivers (photo credit: *GOV.UK *https://www.gov.uk/guidance/a-guide-to-agroforestry).

The aim of this research was to examine UK citizens’ attitudes and preferences associated with implementing agroforestry in rural areas and to explore differences across socio-cultural groups and agronomic contexts. Citizens’ support for three selected types of agroforestry practice (e.g., hedgerows, farm woodlands, or forests along rivers shown in Fig. 1) was also measured. Combining structural equation modeling with latent class analysis, this study is the first to develop a comprehensive model that explains citizens’ attitudes toward agroforestry while identifying and accounting for unobserved heterogeneity among individuals (Sarstedt et al. 2022). It also represents the first national survey to examine citizens’ opinions on implementing agroforestry in rural areas across 12 UK regions. The findings can inform more targeted policy initiatives for future promotion of agroforestry across the UK.

### Agroforestry-specific perceptions and affective response

The literature has examined how perceived benefits and risks associated with innovations aimed at promoting environmental conservation influence citizens’ acceptance. Examples include rewilding land for nature recovery (Titus et al. 2024), creating solar and wind farms to generate renewable energy (Peltonen-Sainio et al. 2020; Scovell et al. 2024), applying biotechnologies to agricultural production and forestry management (Petit et al. 2022; Jin et al. 2023), and planting trees to enhance climate change resilience (Islam et al. 2015; Drew-Smythe et al. 2023). Citizens have been found to associate a variety of benefits with agroforestry. For example, agroforestry has been reported to have aesthetic and recreational value (Gaspar et al. 2016), to improve biodiversity through the provision of wildlife habitat, to improve animal health and welfare (García de Jalón et al. 2018; Bamwesigye et al. 2024), and to be associated with positive productive and economic values (Islam et al. 2015). Citizens also perceive risks related to agroforestry that negatively influence their attitudes toward adoption. These risks include the potential to reduce agricultural productivity and farm income, to increase the complexity of agricultural work, to accumulate anthropogenic waste in the landscape, particularly when public access is granted to visitors, and to cause safety issues (García de Jalón et al. 2018; Torralba et al. 2025).

In addition to deliberate, analytical evaluations of benefits and risks, dual-process theory proposes that individuals also draw on intuition and affect when making decisions (Epstein 1994). Here, “affect” denotes a person’s experiential state of negative to positive feeling. Through learning and experience, different objects, concepts, or images—both perceptual and symbolic—become associated with specific affective states and collectively form an individual’s “affect pool” (Finucane et al. 2000). When an issue is linked to elements within this pool, corresponding emotional responses are triggered. For unfamiliar issues without prior affective associations, the information provided can evoke a situational affect (Siegrist and Sütterlin 2014). The evoked affect then serves as a mental shortcut, guiding judgments and decisions without the need for extensive cognitive processing of the issue (Finucane et al. 2000). Positive affective responses have been associated with higher levels of perceived benefits, lower levels of perceived risks, and more favorable attitudes regarding pro-environmental innovations and behaviors, such as the purchase of green products and participation in waste recycling (Gonçalves et al. 2016; Foroughi et al. 2022). Affect may influence citizens’ decision-making about agroforestry practices, with situational affect playing an important role, given the general lack of familiarity with such practices among citizens (Gao et al. 2014; Gaspar et al. 2016; Mayele and Bongo 2022). Therefore, we propose Hypotheses 1a–e (see Table 1).

**Table 1:** Research hypotheses.

No.		Hypotheses	Evidence
H1	a-c	Positive affect evoked by agroforestry is related to (a) higher benefit perception, (b) lower risk perception, and (c) more positive attitudes regarding agroforestry implementation	Section 1.1
	d	Benefit perception is positively related to attitudes toward agroforestry implementation	
	e	Risk perception is negatively related to attitudes toward agroforestry implementation	
H2	a-c	Greater preference for maintaining the current rural landscape is related to (a) less positive affect, (b) lower benefit perception, and (c) higher risk perception regarding agroforestry implementation	Section 1.2
	d-f	Higher perceived threat to the rural environment is related to (d) more positive affect, (e) higher benefit perception, and (f) lower risk perception regarding agroforestry implementation	
H3	a	Greater preference for maintaining the current rural landscape is related to less positive attitudes toward agroforestry implementation	Section 1.2
	b	Higher perceived threat to the rural environment is related to more positive attitudes toward agroforestry implementation	
H4	a-c	Perceived greater importance of environmental conservation in farming practice is related to (a) more positive affect, (b) higher benefit perception, and (c) lower risk perception associated with agroforestry implementation	Section 1.3
	d-f	Perceived importance of food productivity in farming practice is related to (d) affect, (e) benefit perception, and (f) risk perception regarding agroforestry implementation	
H5	a	Perceived greater importance of environmental conservation in farming practice is related to more positive attitudes toward agroforestry implementation	Section 1.3
	b	Perceived importance of food productivity in farming practice is related to attitudes toward agroforestry implementation	
H6	a-b	Stronger attachment to the countryside is related to (a) greater preference for maintaining the current rural landscape and (b) perceived higher risk to the rural environment	Section 1.4
H7	a-b	Stronger attachment to the countryside is related to perceived greater importance of (a) environmental conservation and (b) food productivity in farming practice	Section 1.4
H8	a-c	Stronger attachment to the countryside is related to (a) more positive evoked affect, (b) higher benefit perception, and (c) lower risk perception regarding agroforestry implementation	Section 1.4
H9		Attachment to the countryside is related to attitudes toward agroforestry implementation, either positive or negative	Section 1.4

### Perceptions and preferences related to the countryside

UK citizens recognize different types of threat to the rural environment, such as climate change and anthropogenic environmental degradation (Stewart-Knox et al. 2024). Those who perceive the rural environment to be at greater risk are more inclined to acknowledge the need to take mitigatory actions and thus tend to hold more positive perceptions and attitudes toward these actions (Drews and van den Bergh 2016; Marikyan and Papagiannidis 2023; Tindale et al. 2023; Stewart-Knox et al. 2024). However, some citizens may resist changes that alter the appearance of the current rural landscapes, even when these changes are the result of pro-environmental initiatives, such as the creation of wind and solar farms (Peltonen-Sainio et al. 2020; Scovell et al. 2024). Therefore, we propose Hypotheses 2a–f and 3a–b (see Table 1).

Agriculture is an important rural industry that plays a significant role in delivering provisioning (e.g., food production), regulating (e.g., water regulation), supporting (e.g., soil fertility), and cultural (e.g., education and recreation) ES (Swinton et al. 2007). At the same time, industrial agriculture exerts negative impacts on rural ecosystems by, for example, reducing the available habitats for wild animals and contributing to soil erosion (Willmott et al. 2023). Public views on farming practices vary due to different perceptions and preferences regarding services delivered by rural ecosystems and different personal values related to farming and land use (El Benni et al. 2024). People who place greater importance on conserving nature and the environment in agricultural production are more likely to hold positive perceptions and attitudes toward pro-environmental farming practices, even when these lead to reduced productivity (Todaro et al. 2023; Herrera et al. 2025). In contrast, those who prioritize productivity and profit in farming often exhibit more negative perceptions and attitudes toward pro-environmental practices, particularly when these practices are perceived to compromise agricultural productivity (Howley et al. 2012; Ives and Kendal 2013). Citizens have been found to associate agroforestry with benefits for nature and the environment (García de Jalón et al. 2018; Bamwesigye et al. 2024), and at the same time to perceive that agroforestry can have positive and negative effects on agricultural production (Islam et al. 2015; García de Jalón et al. 2018; Torralba et al. 2025). Therefore, we propose Hypotheses 4a–f and 5a–b (see Table 1).

### Attachment to the countryside

Place attachment refers to a positive bond a person feels with a specific place (Low and Altman 1992; Jorgensen and Stedman 2001), which involves “an interplay of affect and emotions, knowledge and beliefs, and behaviors and actions in reference to a place” (Low and Altman 1992, p. 5). People with stronger attachment to a place tend to assign greater importance to its various functions and benefits, and to exhibit higher awareness of the threats faced by the place (Jansen 2020; Ganji et al. 2021). This, in turn, may lead to more positive perceptions of, and engagement in, efforts aimed at protecting the place (Song and Soopramanien 2019; Irani et al. 2023). For example, stronger attachment to the countryside has been associated with greater perceived benefits from multiple ES (e.g., food production, biodiversity, and aesthetics), heightened perceptions of environmental risks, and greater support for pro-environmental initiatives in rural areas (Brown and Raymond 2007; Jansen 2020; Gottwald et al. 2022; Parreira and Mouro 2023).

However, conservation efforts sometimes alter the appearance of a place and may be perceived as negative among those with stronger place attachment (Anton and Lawrence 2016). Specifically, changes to the countryside which are perceived to be unnatural (e.g., wind farms) and to disrupt people’s relational values to the countryside may evoke negative societal responses (Devine-Wright and Howes 2010; Daryanto and Song 2021; Irani et al. 2023). Agroforestry practices are unlikely to be perceived as a disruption to people’s attachment to the countryside, as trees are often considered natural changes in rural areas and may contribute to people’s rural attachment (Paniotova-Maczka et al. 2021). Despite this, rapid expansion of agroforestry practices in the countryside may elicit societal concern if planning compromises rural aesthetics, such as through regimented tree planting (Nassauer 1995; Tempesta 2010; Fagerholm et al. 2016). Therefore, we propose Hypotheses 6a–b, 7a–b, 8a–c, and 9 (see Table 1).

**Fig. 2:**
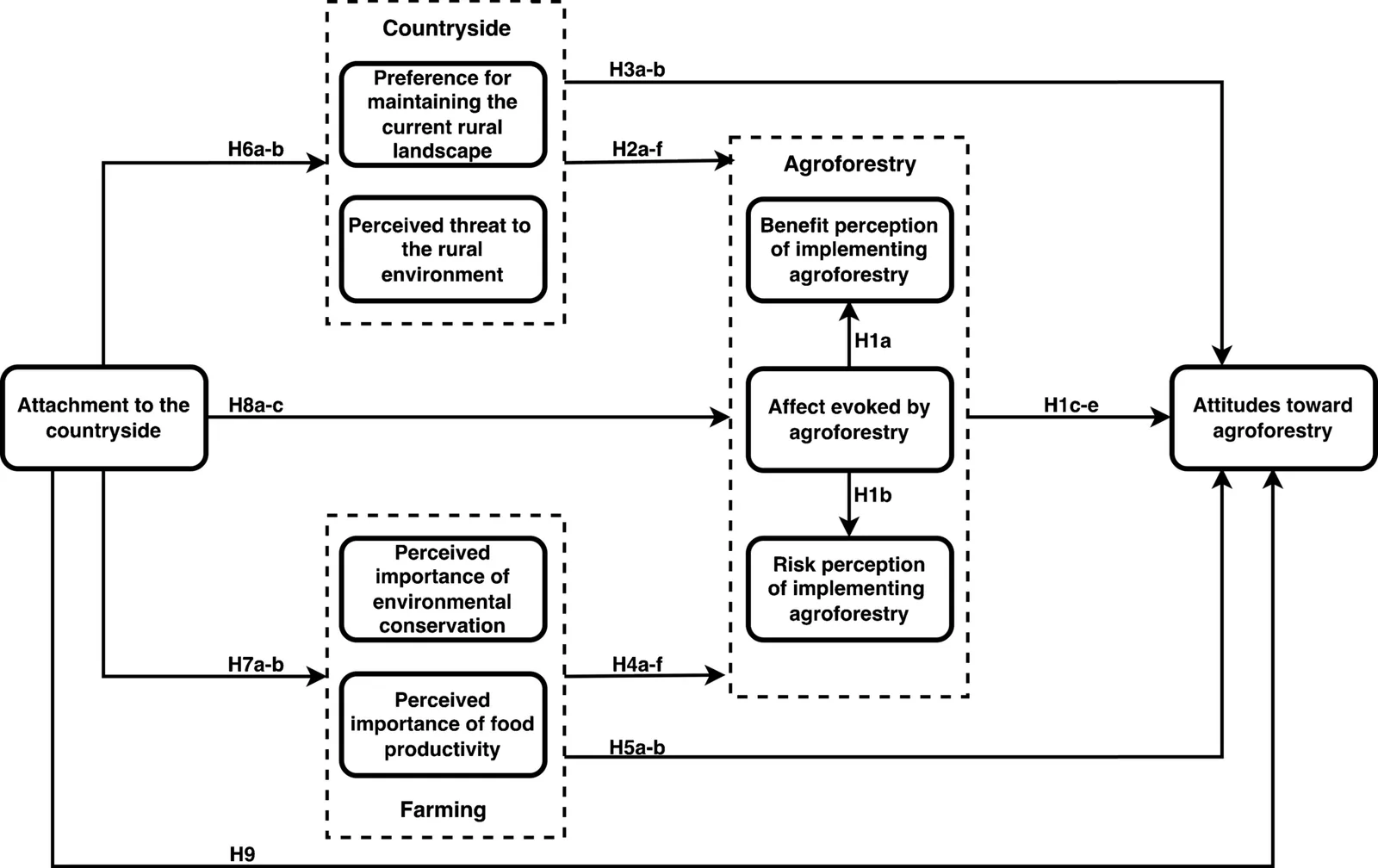
Hypothesized model explaining citizens’ attitudes toward agroforestry.

The hypotheses presented in Table 1 form the basis of the model (Fig. 2), which explains UK citizens’ attitudes toward agroforestry.

## Materials and methods

### Overview of the case study country

Tree planting is a key part of the UK government’s strategy to increase climate resilience and achieve net zero carbon emissions by 2050 (Climate Change Committee 2023). The annual report by the Department for Environment, Food and Rural Affairs and devolved administrations (2024) indicates that agriculture uses more than 70% of the UK’s land and is a major emitter of greenhouse gases (GHGs). Therefore, it may not be possible to meet national tree planting targets and reduce agricultural GHG emissions without converting current agricultural land to alternative land uses such as agroforestry. However, agroforestry systems currently only occupy 3.3% of UK agricultural area, which is less than half the European average (The Woodland Trust 2022). Additionally, UK rural landscapes are perceived to be deeply tied to British heritage, traditions, literature, and national identity (Moore-Colyer and Scott 2005; Shucksmith 2018; Holmes et al. 2022). Changes to rural landscapes, including those induced by agroforestry adoption, may raise societal concerns. Thus, rapid and smooth transition to agroforestry requires effective engagement with a broad range of stakeholders, including not only primary tree growers (such as farmers and land managers) but also citizens, if agroforestry systems and the resulting changes are to be widely accepted (García de Jalón et al. 2018).

### Procedure and sample

An online survey was administered by a social research company (Qualtrics LLC) to UK adults (over 18 years of age) between December 2024 and March 2025 (see Table S1 for items measuring key constructs). Quota sampling based on age, gender, education level, and population proportion in each of the 12 UK regions was used to ensure a nationally representative sample for these demographics (GOV.UK 2024). The following equation was used to determine the required sample size:

$$n=\frac{Z^2p\left(1-p\right)}{E^2}=\frac{{1.96}^2\ast0.5\ast\left(1-0.5\right)}{{0.03}^2}\approx\;1068$$

In this equation, “n” represents the required sample size, “Z” is the value from the standard normal distribution for the selected confidence level, “p” represents standard deviation, and “E” represents the desired margin of error. Using a 95% confidence level (i.e., *Z* = 1.96), an assumed standard deviation of 0.5, and a margin of error of ± 3%, the required minimum sample size was calculated to be 1068 completed surveys. The current study collected 1509 responses, exceeding this minimum requirement. The characteristics of the participants (Table 2) were largely aligned with the UK population in terms of gender, age, and regional distribution. Compared with national statistics, the sample included slightly more individuals with higher education and from North East England, and slightly fewer with upper-secondary education or lower and from Yorkshire and The Humber and South East England (GOV.UK 2024).

**Table 2:** Sample characteristics. *M* denotes mean value; *SD* denotes standard deviation.

	**UK population**	**Sample**	
	**%**	**%**	***n***
***Gender***			
Males	49.0	48.2	728
Females	51.0	51.3	774
Non-binary		0.3	4
Prefer not to say		0.2	3
***Age***		M = 48, *SD* = 17	
18–24 years	10.5	10.3	156
25–34 years	17.0	17.8	268
35–44 years	16.4	17.2	259
45–54 years	16.8	17.5	264
55–64 years	15.9	16.2	245
65 years and older	23.2	21.0	317
***Education level***			
Upper-secondary education or lower	51.0	45.8	691
Tertiary education	49.0	54.2	818
***Region of residence***			
Scotland	8.2	8.1	122
Northern Ireland	2.8	3.2	48
North East	4.0	13.4	202
North West	11.0	10.9	164
Yorkshire and the Humber	8.2	4.4	66
East Midlands	7.4	8.8	133
West Midlands	8.7	8	120
Wales	4.7	4.7	71
East of England	9.3	7.5	113
London	13.5	13.4	202
South West	8.5	9.4	142
South East	13.7	8.3	126

### Survey

Focus group research was conducted to explore public views on tree planting on farmland in the UK (Tindale et al. 2025). The results from this study, along with other published literature (see Supplementary File 1, Table S1), informed the development of the survey. A pilot study was conducted online among 50 UK citizens to further refine the survey questions prior to the main data collection. The survey began with demographic questions and the participants’ self-reported frequency of spending time in the countryside in the past year, followed by items measuring participants’ attachment to the countryside, perceived threat to the rural environment associated with agricultural production, tourism, and climate change, and attitudes toward rural landscape change. Subsequently, participants’ familiarity with the term “agroforestry” and agroforestry practice, affect evoked by, and perceived benefits and risks of, agroforestry, and attitudes toward agroforestry practices were assessed. Finally, values in relation to farming were measured in two dimensions: perceived importance of environmental conservation and productivity in farming practice. The items measuring the constructs included in the proposed model are presented in Table S1. All relevant statements used a 5-point scale ranging from “strongly disagree” to “strongly agree,” except for the evoked affect items, which used a 5-point scale ranging from “extremely negative” to “extremely positive.”

### Data analysis

Descriptive analyses were conducted to provide an overview of the research participants’ perceptions and preferences associated with the countryside and farming as well as agroforestry aspects. The thoughts or images evoked by agroforestry were categorized into different themes, which were associated with different affective states. Responses were mapped to 12 UK regions, and attitudes toward agroforestry implementation across socio-demographic groups were compared using independent *t*-test and Welsh one-way ANOVA. These analyses were performed using IBM SPSS Statistics Version 29 (IBM Corp 2023).

Partial least squares structural equation modeling (PLS-SEM) was performed to test the proposed framework at the whole-sample level using SmartPLS 4 (Ringle et al. 2024). Here, PLS-SEM rather than covariance-based structural equation modeling was selected because the aim of the analysis was to develop a new model for explaining citizens’ attitudes toward agroforestry, rather than to test an existing model (Hair et al. 2017). The following criteria were used to ensure the reliability and validity of the measurement models: Cronbach’s alpha *α* > 0.7 and composite reliability *ρ* > 0.7, values of average variance extracted (AVE) > 0.5, an indicator’s outer loadings on a construct being higher than its outer loadings with other constructs, the application of Fornell-Larcker Criterion (the square root of the AVE of each construct being higher than its highest correlation with any other construct), and the heterotrait-monotrait ratio (HTMT) < 0.9 (Hair et al. 2017). The fit of the structural model was assessed using the following criteria: variance inflation factor (VIF) below 4 to address collinearity between constructs, the significance and relevance of model relationships (T statistics > 1.96 and *p* < 0.05), acceptable coefficients of determination (adjusted R^2^), an exogenous construct’s contribution to an endogenous latent variable’s R^2^ value using the effect size *f*^2^ (the values of 0.02, 0.15, and 0.35 represent a small, medium, and large effect, respectively) (Stone 1974; Rigdon 2012; Hair et al. 2017), and the standardized root mean square residual (SRMR) below 0.08 (Hu and Bentler 1998; Henseler et al. 2014).

**Fig. 3:**
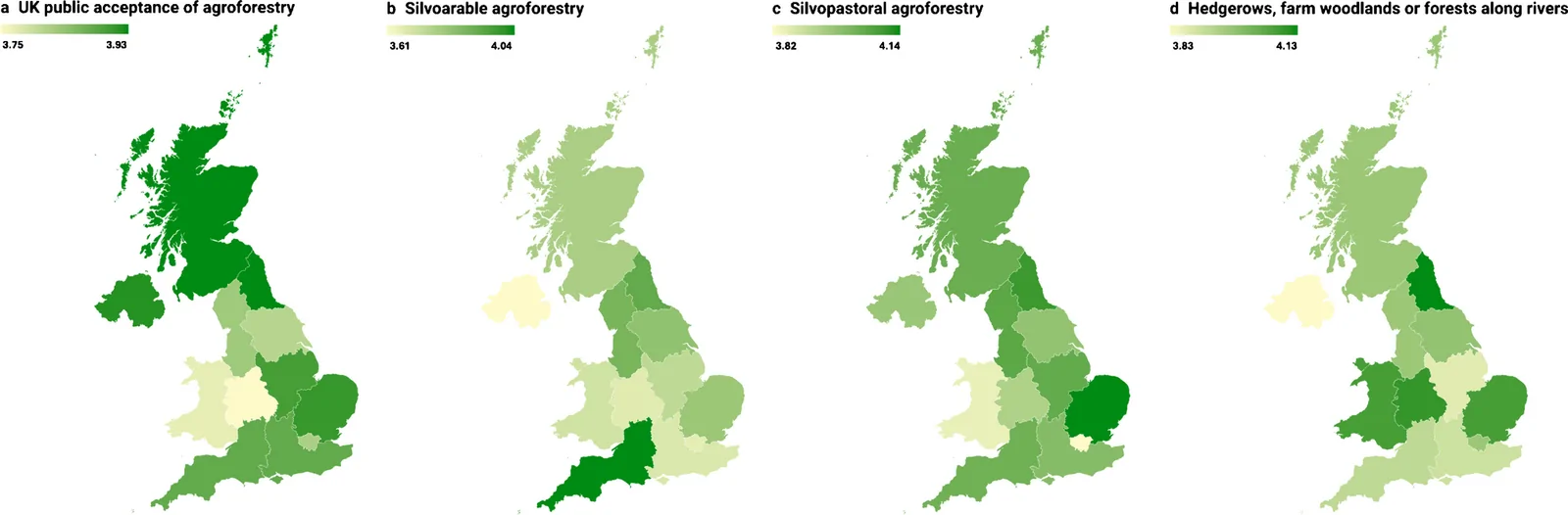
Citizens’ attitudes toward agroforestry in general and toward three specific agroforestry types across 12 UK regions: **a** illustrates UK citizens’ attitudes toward agroforestry in general, while **b**, **c**, and **d** show attitudes toward silvoarable agroforestry, silvopastoral agroforestry, and hedgerows, farm woodlands, or forests along rivers, respectively, across the 12 regions. The greener areas indicate relatively more positive attitudes.

**Fig. 4:**
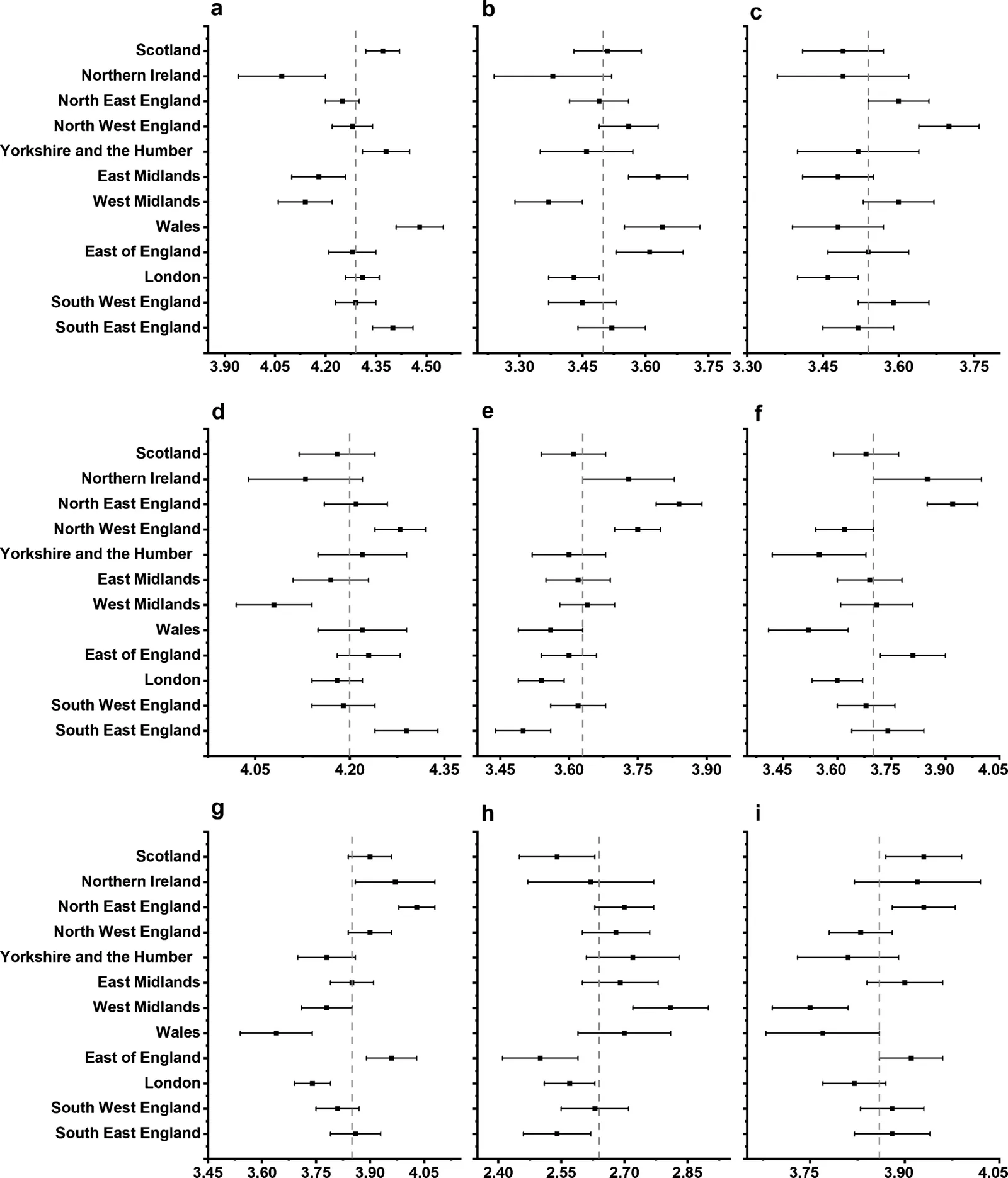
Citizens’ views on the countryside and agroforestry across 12 UK regions: **a** illustrates participants’ attachment to the countryside; **b** shows perceived threat to the rural environment;** c** shows preference for maintaining the current rural landscape;** d** illustrates perceived importance of environmental conservation in farming practice; **e** shows perceived importance of food productivity in farming practice;** f** shows affect evoked by agroforestry; **g** illustrates benefit perception of implementing agroforestry; **h** shows risk perception of implementing agroforestry; and **i** demonstrates attitudes toward agroforestry (1 = strongly disagree; 5 = strongly agree).

To identify and account for unobserved heterogeneity regarding the impacts of different factors on citizens’ attitudes across distinct groups within the sample, a combined approach of latent class analysis and PLS-SEM was employed. This approach included four stages: segmenting the sample based on finite mixture partial least squares (FIMIX-PLS) and partial least squares prediction-oriented segmentation (PLS-POS), assessing measurement models and structural models for each segment (the same criteria as for the whole-sample PLS-SEM), comparing segment-specific effects of the constructs based on permutation multigroup analysis (MGA), and performing an ex post analysis in order to link segments to observable characteristics (Sarstedt et al. 2022). Finally, key findings from all the analyses were mapped across 12 regions of the UK (Figs. 3 and 4).

## Results

### Perceptions related to the countryside and farming

A total of 54.7% of participants reported being frequent visitors to the countryside (visiting more than once a month or having lived there in the past 12 months). Overall, participants demonstrated a strong attachment to the countryside (mean value (*M*) = 4.29, standard deviation (*SD*) = 0.74). The perceived levels of threat to the rural environment (*M* = 3.50, *SD* = 0.89) and preference for maintaining the current rural landscape in environmental management (*M* = 3.55, *SD* = 0.83) ranged from moderate to high. Environmental conservation in farming practice (*M* = 4.20, *SD* = 0.59) was perceived as a highly important criterion by which good farming could be assessed and was rated on average as being more important than food productivity (*M* = 3.64, *SD* = 0.69). Welsh participants indicated the strongest attachment to the countryside and reported greater perceived risk to the rural environment (see Fig. 4). Participants from Northern Ireland showed relatively weaker attachment to the countryside compared to other regions, and participants from West Midlands perceived the lowest threat to the rural environment. Those from North West England expressed the highest preference for maintaining the current rural landscape within environmental management initiatives, and those from London showed the lowest preference (Fig. 4). Overall, participants across the 12 UK regions perceived environmental conservation to be more important than food productivity in farming. Those from South East England perceived the highest importance of environmental conservation and lowest importance of food productivity in farming. Those from West Midlands perceived the lowest importance of care for nature in farming, and those from North East England perceived the highest importance of food productivity in farming.

### Familiarity with and affective response to agroforestry

A total of 46.1% of participants reported being aware prior to the survey that farmers integrate trees and shrubs into their practices; however, only 30.1% were familiar with the specific term “agroforestry.” A brief description of agroforestry was subsequently shown to participants (see Table S1), who were then asked what the first thought or image was that they associated with the word “agroforestry” (open-ended question) and whether their first thought/image was positive or negative. The responses suggest that the term agroforestry evoked various thoughts and images, which were generally associated with neutral to positive feelings. The thoughts and images evoked in participants’ minds were coded into six main thematic categories, representing approximately 96% of the responses. “Trees and forest” and “unfamiliarity” were the two most common categories of thoughts or images, accounting for 37.9% and 31.9% of the responses. They were associated with moderately positive (*M* = 3.90, *SD* = 0.92) and neutral to slightly positive (*M* = 3.41, *SD* = 0.97) feelings, respectively. “Risk and uncertainty” accounted for 7.7% of the responses and tended to be associated with negative feelings (*M* = 2.73, *SD* = 1.04). “Land management” accounted for 7.5% of the responses, “conservation of nature and the environment” for 6.7%, and “land-use change” for 4.9%. These three categories (“land management,” “conservation of nature and the environment,” and “land-use change”) were associated with positive feelings (average affect value > 4).

### Perceptions and attitudes toward agroforestry

In general, agroforestry was perceived to provide a moderate to high level of benefit (*M* = 3.86, *SD* = 0.74) and pose a low to moderate level of risk (*M* = 2.64, *SD* = 0.96). Benefits concerning habitat creation for wildlife, greenhouse gas capture, and flood control on farmlands were rated most highly. Negative socio-economic consequences, such as delayed benefit realization, higher input requirements, and potential conflicts among stakeholders, were identified as representing the greatest risks (for full details of perceived benefits and risks see Supplementary File 1, Table S2). Participants exhibited generally neutral to positive attitudes toward agroforestry (*M* = 3.86, *SD* = 0.67), with 57.3% expressing “positive” attitudes (i.e., attitude values of 4 or higher on a 5-point scale). Across the 12 UK regions (see Fig. 4), agroforestry evoked the most positive feelings among participants in South West England, who also reported the highest benefit perceptions. In contrast, participants in London reported the least positive feelings and the lowest benefit perceptions. In Northern Ireland, participants reported the highest perceived risks associated with agroforestry, and those in North West England reported the lowest. Participants in North East England and Scotland had more positive attitudes toward agroforestry, and those in the West Midlands had less positive attitudes compared to other regions.

In terms of the three different types of agroforestry (for the agroforestry photos used to elicit responses, see Table S1), participants showed moderate to high levels of support for silvoarable agroforestry (*M* = 3.79, *SD* = 0.86), silvopastoral agroforestry (*M* = 4.01, *SD* = 0.81), and hedgerows, farm woodlands, or forests along rivers (*M* = 3.96, *SD* = 0.87). A repeated measures ANOVA with a Greenhouse-Geisser correction determined that the levels of participants’ support differed significantly between agroforestry types (*F*(1.912, 2883.2) = 56.911, *p* < 0.001). Post hoc analysis with a Bonferroni adjustment revealed that participants’ support for implementing silvopastoral agroforestry (0.22 (95% CI, 0.168 to 0.272), *p* <.001) and hedgerows, farm woodlands or forests along rivers (0.178 (95% CI, 0.120 to 0.235), *p* <.001) was significantly higher compared to implementing silvoarable agroforestry. This pattern was consistent across most UK regions (see Table 3). The exception was South West England, where silvoarable and silvopastoral agroforestry received higher levels of support compared with hedgerows, farm woodlands, or forests along rivers. Of the 12 regions (Fig. 3), implementing silvoarable agroforestry received the highest level of support in South West England, and the lowest in Northern Ireland. Silvopastoral agroforestry received the highest level of support in East of England, and the lowest in London. Hedgerows, farm woodlands, or forests along rivers received the highest level of support in North East England and the lowest in Northern Ireland.

**Table 3:** Citizen attitudes toward three types of agroforestry practice. *M* denotes mean value; *SD* denotes standard deviation. Attitudes toward agroforestry refer to support for introducing a specific type of agroforestry on UK farms (1 = strongly disagree; 5 = strongly agree).

**Regions**	**Silvoarable**	**Silvopastoral**	**Hedgerows, woodlands, or forests along rivers**
	*M* (*SD*)		
Scotland	3.78 (0.82)	4.04 (0.78)	3.97 (0.78)
Northern Ireland	3.61 (0.99)	3.97 (0.76)	3.83 (0.87)
North East England	3.92 (0.82)	4.10 (0.72)	4.13 (0.79)
North West England	3.88 (0.79)	4.06 (0.78)	3.97 (0.78)
Yorkshire and The Humber	3.83 (0.84)	3.98 (0.79)	3.98 (0.94)
East Midlands	3.74 (0.86)	4.05 (0.88)	3.87 (0.95)
West Midlands	3.67 (0.90)	3.94 (0.88)	4.10 (0.85)
Wales	3.70 (0.80)	3.85 (0.79)	4.08 (0.81)
East of England	3.81 (0.86)	4.14 (0.80)	4.08 (0.79)
London	3.66 (0.86)	3.82 (0.88)	3.97 (0.91)
South West England	4.04 (0.83)	4.03 (0.85)	3.92 (0.95)
South East England	3.68 (0.85)	4.00 (0.75)	3.90 (0.83)

Responses across socio-demographic groups were analyzed. An independent *t*-test showed that participants who had attained tertiary education (*M* = 3.95, *SD* = 0.64) had significantly more positive attitudes toward agroforestry compared to participants who did not fall into this group ((*M* = 3.76, *SD* = 0.68), *t*(1,507) = 5.54, *p* < 0.001). Similarly, frequent visitors to the countryside (*M* = 3.92, *SD* = 0.66) had significantly more positive attitudes toward agroforestry compared to others ((*M* = 3.79, *SD* = 0.67), *t*(1,507) = 3.93, *p* < 0.001). Participants aged 25–34 (*M* = 3.95, *SD* = 0.67) had the most positive attitudes toward agroforestry, while those aged 18–24 years had the least positive attitudes (*M* = 3.73, *SD* = 0.71). Given the unequal sample sizes across age groups, Welsh one-way ANOVA and the Games-Howell post hoc test were used to compare participants’ attitudes. The results showed that participants aged 25–34 and 55–64 years (*M* = 3.93, *SD* = 0.62) had significantly more positive attitudes toward agroforestry compared to those aged 18–24 years. No gender differences in attitudes toward agroforestry were found.

### Results of whole-sample SEM

All reliability and validity criteria for the measurement and structural models were met (see Supplementary File 1, Table S1 and S3). The resulting model showed no critical collinearity issues between the assessed constructs (VIF < 5) and had a good fit (SRMR = 0.05). The model explained 65% of the variance in participants’ attitudes toward implementing agroforestry in the countryside (*R*^*2*^ = 0.65), indicating substantial explanatory power. Direct effects between constructs, including standardized values of path coefficients β, the respective T-statistics, 95% confidence intervals, and F^2^ are presented in Table 4. The results showed that individuals for whom agroforestry evoked more positive affect also reported lower risk perception, higher benefit perception, and more positive attitudes regarding agroforestry, supporting all H1 hypotheses. Participants who reported greater preferences for maintaining the current rural landscape perceived higher risks to be associated with agroforestry. As such, H2c was supported. Participants who perceived higher risk to the rural environment reported more positive affect evoked by agroforestry, together with higher benefit perception of, and more positive attitudes toward, agroforestry, supporting H2d–e and H3b. Placing high importance on environmental conservation and food productivity in farming was found to be associated with more positive affect evoked by agroforestry (H4a and 4 d supported) and attitudes toward agroforestry (H5a–b supported). The perceived importance of food productivity was also associated with higher risk perception related to agroforestry (H4f supported). Participants with a stronger attachment to the countryside had higher levels of perceived environmental risk in rural areas, greater preference for maintaining the current rural landscape, and perceived greater importance of environmental conservation and food productivity in farming practice, supporting H6 and H7. As predicted, stronger attachment to the countryside was associated with more positive affect evoked by agroforestry (H8a supported) and lower risk perceptions related to agroforestry (H8c supported).

**Table 4:** Estimation results of the model. *, **, and *** indicate significance at *p* < 0.05, 0.01, and 0.001, respectively; β denotes path coefficients; *SD* denotes standard deviation; 95% CI denotes 95% confidence interval; *f*^2^ denotes the effect size.

Hypotheses		***β***	***SD***	T statistics	95% CI	***f***^2^
H1a	Affect -> Benefit perception of agroforestry	0.402***	0.025	16.305	[0.351, 0.448]	0.234
H1b	Affect -> Risk perception of agroforestry	−0.282***	0.026	11.032	[−0.331, −0.232]	0.088
H1c	Affect -> Attitudes toward agroforestry	0.188***	0.02	9.264	[0.148, 0.227]	0.070
H1d	Benefit perception of agroforestry -> Attitudes toward agroforestry	0.572***	0.026	22.29	[0.521, 0.622]	0.513
H1e	Risk perception of agroforestry -> Attitudes toward agroforestry	−0.112***	0.019	6.001	[−0.149, −0.076]	0.026
H2a	Preference for maintaining current rural landscape -> Affect	0.017	0.028	0.602	[−0.038, 0.070]	0.000
H2b	Preference for maintaining current rural landscape -> Benefit perception of agroforestry	0.031	0.023	1.347	[−0.015, 0.075]	0.001
H2c	Preference for maintaining current rural landscape -> Risk perception of agroforestry	0.140***	0.025	5.539	[0.089, 0.188]	0.023
H2d	Perceived threat to the rural environment -> Affect	0.060*	0.025	2.372	[0.012, 0.110]	0.004
H2e	Perceived threat to the rural environment -> Benefit perception of agroforestry	0.101***	0.023	4.41	[0.056, 0.146]	0.015
H2f	Perceived threat to the rural environment -> Risk perception of agroforestry	0.097***	0.027	3.56	[0.043, 0.150]	0.011
H3a	Preference for maintaining current rural landscape -> Attitudes toward agroforestry	−0.02	0.016	1.259	[−0.051, 0.011]	0.001
H3b	Perceived threat to the rural environment -> Attitudes toward agroforestry	0.036*	0.018	2.043	[0.002, 0.070]	0.003
H4a	Perceived importance of environmental conservation -> Affect	0.236***	0.029	8.205	[0.179, 0.293]	0.047
H4b	Perceived importance of environmental conservation -> Benefit perception of agroforestry	0.298***	0.027	11.12	[0.246, 0.350]	0.103
H4c	Perceived importance of environmental conservation -> Risk perception of agroforestry	−0.307***	0.026	11.937	[−0.358, −0.257]	0.084
H4d	Perceived importance of food productivity -> Affect	0.154***	0.027	5.798	[0.102, 0.206]	0.024
H4e	Perceived importance of food productivity -> Benefit perception of agroforestry	0.031	0.024	1.288	[−0.015, 0.079]	0.001
H4f	Perceived importance of food productivity -> Risk perception of agroforestry	0.212***	0.028	7.605	[0.159, 0.267]	0.049
H5a	Perceived importance of environmental conservation -> Attitudes toward agroforestry	0.059**	0.023	2.508	[0.013, 0.105]	0.006
H5b	Perceived importance of food productivity -> Attitudes toward agroforestry	0.081***	0.017	4.666	[0.047, 0.115]	0.015
H6a	Attachment to the countryside -> Preference for maintaining current rural landscape	0.191***	0.026	7.363	[0.141, 0.241]	0.038
H6b	Attachment to the countryside -> Perceived threat to the rural environment	0.166***	0.028	5.943	[0.113, 0.222]	0.028
H7a	Attachment to the countryside -> Perceived importance of environmental conservation	0.395***	0.028	14.114	[0.341, 0.451]	0.184
H7b	Attachment to the countryside -> Perceived importance of food productivity	0.150***	0.027	5.582	[0.097, 0.203]	0.023
H8a	Attachment to the countryside -> Affect	0.062*	0.028	2.197	[0.007, 0.119]	0.004
H8b	Attachment to the countryside -> Benefit perception of agroforestry	0.033	0.023	1.459	[−0.011, 0.077]	0.001
H8c	Attachment to the countryside -> Risk perception of agroforestry	−0.063**	0.025	2.562	[−0.113, −0.016]	0.004
H9	Attachment to the countryside -> Attitudes toward agroforestry	−0.003	0.017	0.157	[−0.037, 0.032]	0.000

To better understand the impacts of different constructs on attitudes toward agroforestry, indirect and total effects were also calculated (Table S4). In terms of direct effects on attitudes toward agroforestry, benefit perception was the most influential on participants’ attitudes, followed by affect and risk perception. The perceived importance of environmental conservation and food productivity in farming practice and perceived risk to the rural environment also showed positive direct effects. Participants’ preference for maintaining the current rural landscape and attachment to the countryside had no significant direct effects. After taking indirect effects into account, benefit perception and evoked affect remained the two most influential constructs. However, the effects of the perceived importance of environmental conservation and food productivity in farming practice and attachment to the countryside were all greater than the effect of risk perception, with the perceived importance of environmental conservation being the most influential. Participants’ perceived risk to the rural environment had a similar level of effect on attitudes to the effect of risk perception associated with agroforestry, while preference for maintaining the current rural landscape had no indirect effect on attitudes.

### Results of the latent class analysis

Using FIMIX-PLS, three segments were selected as the optimal solution for the data (Table S5). PLS-POS, a hill-climbing approach that gradually reallocates objects between the segments to maximize the solution’s weighted *R*^2^, was then used to run segmentation using a three-segment solution. PLS-POS achieved three segments which accounted for 27.6% (*n* = 417), 31.1% (*n* = 469), and 41.3% (*n* = 623) of the sample. The measurement and structural models across the three segments met all the reliability and validity criteria (Table S6). Detailed procedures can be found in Supplementary File 2.

Each of the identified segments was named based on its difference from the other two segments in terms of the effects of the constructs on participants’ attitudes toward agroforestry. The first segment (S1) was named “cautious conservation-oriented citizens,” whose attitudes toward agroforestry were more positively influenced by perceived importance of environmental conservation while also more negatively affected by risk perception of agroforestry. The second segment (S2) was named “citizens sensitive to threats to the rural environment,” for whom affect and attitudes associated with agroforestry were positively affected by perceived risk to the rural environment, to a significantly greater extent compared to S1 and S3. The third segment (S3) was named “countryside-engaged eco-productive citizens,” whose attitudes toward agroforestry were largely driven by attachment to the countryside, as well as the perceived importance of both environmental conservation and high productivity in farming. Table S4 shows the direct and indirect impacts of the constructs on attitudes toward agroforestry implementation across the three segments. Detailed multigroup analyses and results can be found in Supplementary File 1, Table S7.

Ex post analyses were conducted to compare the mean values of the constructs included in the model across three segments (Table S8). The results of the Welsh one-way ANOVA and the Games-Howell post hoc test indicated that participants in S3 had significantly greater preference for maintaining the current rural landscape compared to those in S2 and placed greater importance on food productivity in farming than the other two segments. Agroforestry evoked significantly more positive affect among participants in S1 compared to S2. Participants in S3 had the highest risk perception of agroforestry, followed by S2 and S1, and held significantly more positive attitudes than those in S2. There was a significant difference across segments regarding participants’ age (*M* = 49 for S1 and S2 and *M* = 46 for S3), with the average age for S3 significantly younger than S2. The results of a multinomial logistic regression indicated that participants who had completed higher education were more likely to belong to S3 compared to S2. The results of Chi-square showed that participants across segments had no significant differences regarding gender distribution or whether they were frequent countryside visitors. The proportions of participants belonging to the three citizen segments varied across the 12 UK regions (Table S9). The highest percentages were observed in London for S1 (33.7%), the West Midlands for S2 (40.0%), and Northern Ireland for S3 (52.1%). The lowest percentages were found in Wales for S1 (19.7%), the North East England for S2 (24.8%), and London for S3 (35.1%). Additionally, some researchers suggested using a combination of several variables to create groupings that largely correspond to the latent segments obtained through PLS-POS. An overlap of 60% between the PLS-POS partition and the one produced by the explanatory variables is considered satisfactory (Matthews et al. 2016; Sarstedt et al. 2022). Based on a multinomial logistic regression, we used four variables which successfully predicted 67.1%, 70.1%, and 80.7% of the cases for S1, S2, and S3, respectively. Detailed analysis can be found in Table S10.

## Discussion

Using data from a representative UK sample, we find that participants perceive the benefits of agroforestry as outweighing its risks to the countryside and hold overall positive attitudes toward it. These findings are consistent with the research conducted in Germany, India, and Uganda (Islam et al. 2015, 2017; Otter and Langenberg 2020; Bamwesigye et al. 2024). However, regional differences in citizens’ perceptions and attitudes related to agroforestry exist in the UK. These differences should be further explored and considered in policymaking since tree planting proposals in regions where stakeholder views are overlooked have provoked negative societal responses. For example, the 2024 update of the Sustainable Farming Scheme required Welsh farmers to plant trees on 10% of their land in order to qualify for government funding, which triggered widespread protests (Messenger 2024). Although this requirement had been dropped by the time of this study, the protests may have negatively influenced public support for agroforestry. This could explain why participants from Wales reported the lowest perceived benefits of, and the second least positive attitudes toward, agroforestry. In contrast, North East England has a historical tradition of upland pastoral farming and open landscapes, alongside recent citizen engagement in government-led tree and hedgerow planting initiatives (BBC News 2022, 2024). These factors may have contributed to participants from the North East expressing the highest perceived benefits of, and the most positive attitudes toward, agroforestry.

To our knowledge, this study is the first to develop and test a comprehensive model that explains citizens’ attitudes toward agroforestry. The model demonstrates substantial explanatory power (*R*^*2*^ = 0.65) and generates new insights into attitude formation by considering both the direct and indirect impacts of key factors, as well as the heterogeneity across three latent segments within the sample. Existing quantitative studies on public attitudes toward agroforestry have primarily focused on its direct impacts (Otter and Langenberg 2020; Bamwesigye et al. 2024), potentially overlooking indirect effects that could play a significant role in enhancing public support for agroforestry. In our study, when indirect impacts were taken into account, the perceived importance of environmental conservation, food productivity, and attachment to the countryside had greater total effects on public attitudes than the perception of agroforestry-related risks. Furthermore, the total effects of different factors on attitudes suggested heterogeneity across the three segments, which resonates with divergent environmental worldviews and identities. Specifically, *cautious conservation-oriented citizens* (S1) reflect a transition from human-centered values toward an eco-centric perspective, but remain cautious about potential land-use changes brought by agroforestry (Thompson and Barton 1994; Frantz et al. 2025). In contrast, *countryside-engaged eco-productive citizens* (S3) represent a hybrid worldview that rejects the binary choice between production and protection, mirroring a reformist environmentalism that seeks to green industrial systems from within without necessitating a radical restructuring of existing human-nature relationships (Devall 1979; Hernández and Muñoz 2022). *Citizens sensitive to threats to the rural environment* (S2) reflect a defensive rural environmental stewardship identity. In this segment, new land-use developments in rural areas, including agroforestry, can receive support only if they are perceived to be capable of mitigating existing threats to the rural environment (Devine‐Wright 2009; Flood et al. 2022). Accounting for both the direct and indirect effects of influencing factors, as well as their varied impacts across different population segments, is important when designing targeted interventions to foster public support for agroforestry.

Another important contribution of our study is an enhanced understanding of how place attachment shapes attitudes toward agroforestry. Rather than directly influencing perceptions and attitudes, attachment to the countryside shaped attitudes toward agroforestry mainly by influencing perceptions and preferences associated with the countryside and farming. Participants who reported greater attachment to the countryside expressed a higher level of concern about risks to the rural environment and placed higher importance on environmental conservation in farming practices. This in turn was found to be associated with higher levels of perceived benefits from agroforestry, leading to more positive attitudes toward the practice. However, attachment to the countryside was also positively correlated with a preference for maintaining the current rural landscape and greater perceived importance of food production, with an increased perception of risk associated with agroforestry implementation. The effects of attachment to the countryside varied across the three segments. As such, although previous research suggested that place attachment has the potential to bridge geographic and social boundaries and could be leveraged to foster pro-environmental attitudes and behaviors (Gurney et al. 2017), place attachment may also lead to conflict over agroforestry implementation, especially in regions where attachment to the countryside is particularly strong. The different impact pathways of place attachment and the observed differences across segments may explain why place attachment sometimes has no or limited positive impact on citizens’ benefit perception of, and support for, specific pro-environmental initiatives (e.g., see Bonaiuto et al. 2016; Groshong et al. 2020; Paniotova-Maczka et al. 2021), or even a negative impact, especially for initiatives perceived to represent threats to local identity, landscape, and food production (e.g., see Bonaiuto et al. 2016; Devine-Wright and Howes 2010).

### Policy implications for agroforestry promotion

Our results have important policy implications that can help the design of future agroforestry schemes to maximize benefits for society and reduce conflict between stakeholder groups. First, citizens’ preferences and priorities associated with agroforestry should be taken into consideration when developing policies and interventions aiming to promote agroforestry. We find that citizens value wildlife habitat creation and climate change resilience and are moderately concerned about the use of exotic species. In response, policy and incentive schemes should prioritize “good” agroforestry practices, such as planting native tree species and crops following agroecological principles. This approach favors ecological integrity over “agrobizforestry” or “agrodeforestry,” models that risk reinforcing the industrial plantation economy over the long term (Ollinaho and Kröger 2021). Citizens’ values and preferences should be analyzed along with farmers’ and other stakeholders’ preferences to identify areas of conflict and agreement (Fu et al. 2025). For example, farmers in South East England and East of England were previously found to prefer silvoarable agroforestry over silvopastoral agroforestry (Felton et al. 2023), whereas citizens in these regions showed the opposite preference in our study. In regions where views between citizens and farmers diverge more strongly, there may be increased citizen-farmer tensions regarding agroforestry implementation, thereby impeding efforts to increase agroforestry creation (Iversen et al. 2022). This highlights the need for enhanced communication through active engagement and co-creation activities, with the aim of fostering mutual understanding, potentially reaching consensus, and co-designing future agroforestry schemes.

Second, differences across citizen segments should be considered in order to inform more effective and targeted communication. To gain greater citizen support for implementing agroforestry, activities and actions that have been made to address citizens’ preferences and priorities in policy and practice should be communicated. Introducing agroforestry in ways that evoke more positive affect—such as through framing and imagery aligned with citizens’ values and preferences—may further enhance citizens’ receptiveness. This is particularly important for regions where there are more participants belonging to S1 (*cautious conservation-oriented citizens*) and S2 (*citizens sensitive to threats to the rural environment*), since affect plays an important role in shaping attitudes toward agroforestry among these segments. Scientific evidence on the environmental and agricultural impacts of agroforestry should also be communicated to citizens. This may be particularly important in regions such as Northern Ireland and North East England, where affective responses were less influential on citizens’ attitudes for a larger proportion of participants (S3: *countryside-engaged eco-productive citizens*). In such areas, locally tailored public engagement activities, such as public exhibitions that present the histories, current realities, and possible futures of local agroforestry landscapes, may be especially effective in fostering knowledge exchange (National Landscape Discovery Centre 2025). However, environmental worldviews may change over time—for instance, following critical environmental experiences (such as climate change events) or policy shifts, resulting in citizens moving between existing segments or forming entirely new ones (Arcury and Christianson 1990; Dunlap and York 2008). Consequently, strategies promoting agroforestry and other land-use practices in rural areas should be continuously reassessed to align with these evolving worldviews.

Third, a more holistic perspective that considers complex relationships between the influencing factors is needed when developing interventions aimed at increasing citizen acceptance of agroforestry. Research has suggested that strengthening place attachment can increase citizens’ support for pro-environmental actions (Groshong et al. 2020; Daryanto and Song 2021). However, in the context of promoting agroforestry, this may lead to unintended effects, such as increased citizen concern about potential changes to current rural landscapes and the resulting higher risk perception associated with agroforestry. At present, agroforestry-related policies in the UK do not adequately address land-use changes at the landscape level, which may lead to piecemeal or culturally discordant transformations of the countryside. Therefore, the UK government should support farmers in coordinating with their neighbors and aligning with regional landscape plans before implementing agroforestry practices. When developing interventions to strengthen citizens’ countryside attachment, it is important to address these challenges—for example, by using virtual reality technology to show how agroforestry creation can change the landscape in ways that reflect citizens’ preferences.

### Research limitations and recommendations

The attitudes of UK citizens toward agroforestry were assessed using an online survey administered to a nationally representative sample. However, individuals with limited internet access may have been excluded. Future research should actively engage these marginalized people to ensure a more inclusive and comprehensive analysis of citizen responses to agroforestry implementation. Regarding research design, a minimal, “information-lean” prompt was employed to elicit baseline public attitudes while minimizing the potential for framing effects induced by researcher-led information provision. The validity of this approach is supported by the finding that respondents prioritized wildlife habitat creation over climate-related benefits, even though “climate change” served as the primary frame in the survey introduction. This finding suggests that the responses were not merely an artifact of the prompt’s phrasing. However, we acknowledge that the answers of respondents previously unfamiliar with the term “agroforestry” or the practice of integrating trees on farmland might be speculative. Future research could look into the role of information by segmenting respondents based on prior familiarity and employing split-sample information experiments. Such experiments would systematically compare baseline perceptions with attitudes formed after exposure to diverse information messages (e.g., benefit-led, risk-oriented, or balanced). These messages should refer to both short- and long-term impacts while differentiating between different models of implementation, ranging from socio-ecologically “good” agroforestry to more industrial “agrobizforestry” or “agrodeforestry” paradigms. In terms of data analysis, the correlations in this research should not be assumed to indicate causation. Experimental methods can explore the causal relationships between the variables of interest in future research. Future research could also experimentally explore affective responses to agroforestry-driven transformations. For instance, virtual reality could simulate changes to, and the potential reconciliation of, various ES associated with agroforestry (Rüegg et al. 2025), such as productivity, carbon sequestration, metabolic flows, biodiversity, and landscape aesthetics, under different policy scenarios.

## Conclusions

To the best of the authors’ knowledge, this study is the first to develop and test a comprehensive hybrid model explaining UK citizens’ attitudes toward agroforestry while accounting for unobserved heterogeneity in the effects of key factors across three distinct groups within the sample. Evoked affect and benefit perceptions were two agroforestry-specific factors that consistently played important roles in shaping attitudes toward agroforestry. However, combined with latent class analysis, the modeling also highlighted the importance of perceptions related to the countryside and farming. For instance, the perceived importance of environmental conservation in farming exhibited an even greater positive influence on attitudes than benefit perceptions among *cautious conservation-oriented citizens.* Furthermore, both the perceived importance of food productivity and that of environmental conservation in farming demonstrated greater positive effects on attitudes than evoked affect among *countryside-engaged eco-productive citizens*. By analyzing both overall and segment-specific interrelationships among the factors included in the model, this study offers valuable policy implications to facilitate future agroforestry promotion.

## Supplementary Information

Below is the link to the electronic supplementary material.

ESM 1 (DOCX 120 KB)

ESM 2 (DOCX 28.3 KB)

## Data Availability

The data is available at: 10.5281/zenodo.20507440.
