# Supplementary material for: Driving climate resilience: citizen attitudes toward agroforestry and their policy implications in the UK
Source: Agron Sustain Dev. 2026 Jul 13;46(4):53. doi: 10.1007/s13593-026-01130-w (PMC13364824; doi:10.1007/s13593-026-01130-w)
Supplement: Supplementary file 1 — (DOCX 120 KB) [file 13593_2026_1130_MOESM1_ESM.docx]

**Supplementary File 1**

**Table S1.** Broad perceptions related to the countryside and farming and agroforestry-specific response

| **Construct** | **Item** | **References** | **Mean (*SD*)** | **Cronbach’s α** | **CR** | **AVE** |
| --- | --- | --- | --- | --- | --- | --- |
| **Attachment to the countryside** | I feel relaxed when I am in the countryside. I feel happy when I am in the countryside. I like to be in the countryside.  I really miss the countryside when I have not been there for a long time. | (Jorgensen and Stedman, 2001) | 4.29 (0.74) | 0.885 | 0.886 | 0.745 |
| **Perceived  threat to the rural environmental** | I’m worried about the negative impacts on the environment in the countryside caused by agricultural production. I’m worried about the negative impacts on the environment in the countryside caused by visitors and tourists. I’m worried about the negative impacts on the environment in the countryside caused by climate change. | (Stewart-Knox et al., 2024) | 3.50 (0.89) | 0.753 | 0.837 | 0.661 |
| **Preference for maintaining the current rural landscape** | The appearance of the countryside should remain unchanged when taking actions to mitigate the negative impacts of climate change The appearance of the countryside should remain unchanged when taking actions to increase the countryside’s resilience to climate change Changes to the appearance of the countryside will negatively impact on people’s local identities and ways of life | (Peltonen-Sainio et al., 2020; Wheeler, 2017) | 3.55 (0.83) | 0.812 | 0.814 | 0.728 |
| **Affect evoked by agroforestry** | (Brief introduction: *In response to the impacts of climate change on the countryside and beyond, the UK government has made a series of plans, including the promotion of agroforestry (trees integrated into farming) across the country*.)  **a.** What is the first thought or image that comes to your mind after you read the word “agroforestry”? **b.** Is your first thought or image of agroforestry positive or negative? | (Jin et al., 2022; Leiserowitz, 2006) | 3.70 (1.02) | NA | NA | NA |
| **Benefit perception** | Agroforestry is beneficial for me personally. Agroforestry is beneficial for the environment. Agroforestry is beneficial for the UK countryside. Agroforestry is beneficial for UK society as a whole. | (Fischer et al., 2013; Jin et al., 2022) | 3.86 (0.74) | 0.896 | 0.901 | 0.764 |
| **Risk perception** | Agroforestry could have a negative impact on me personally. Agroforestry could have a negative impact on the environment. Agroforestry could have a negative impact on the UK countryside. Agroforestry could have a negative impact on UK society as a whole. | (Jin et al., 2022; Wilson et al., 2019) | 2.64 (0.96) | 0.923 | 0.925 | 0.812 |
| **Attitudes towards agroforestry** | Agroforestry is a good thing. Agroforestry on UK farms is appropriate. I support introducing agroforestry on UK farms | (Fischer et al., 2013; Jin et al., 2022) | 3.86 (0.67) | 0.881 | 0.881 | 0.808 |
| **Attitudes towards silvoarable agroforestry** | ***Introduction:*** Silvoarable agroforestry (the integration on a parcel of land of arable crops and trees)  ***Item:*** I support introducing this type of agroforestry on UK farms | The photo used in our survey is the first image in the “SILVOARABLE” section of the following webpage: *Regenerative Food & Farming*: <https://regenerativefoodandfarming.co.uk/agroforestry/>. | 3.79 (0.86) | NA | NA | NA |
| **Attitudes towards silvopastoral agroforestry** | ***Introduction:*** Silvopastoral agroforestry (combining trees or shrubs with livestock and pasture production on the same unit of land)  ***Item:*** I support introducing this type of agroforestry on UK farms | The photo used in our survey is the image right next to the content about “silvopasture” of the following webpage: *Woodland Trust*: <https://www.woodlandtrust.org.uk/plant-trees/agroforestry-benefits/>. | 4.01 (0.81) | NA | NA | NA |
| **Attitudes towards hedgerows, farm woodlands or forests along rivers** | ***Introduction:*** Hedgerows, farm woodlands or forests along rivers  ***Item:*** I support introducing this type of agroforestry on UK farms | The photo used in our survey see Fig. 1; photo from GOV.UK: <https://www.gov.uk/guidance/a-guide-to-agroforestry>. | 3.96 (0.87) | NA | NA | NA |
| **Perceived importance of environmental conservation as a criterion for good farming** | A good farmer is one that… Maintains or increases soil organic matter. Considers health of streams on their land. Manages for both profit and environment. Considers long-term conservation of farm resources before short-term profits. Maintains habitat for wildlife. Minimises the use of pesticides/fungicides that harm wildlife health. Minimises negative impacts of farming on wildlife. | (Dixon et al., 2022) | 4.20 (0.59) | 0.892 | 0.893 | 0.606 |
| **Perceived importance of food productivity as a criterion for good farming** | A good farmer is one that... Uses new technologies in farming. Has up-to-date equipment. Maximises farming income. Produces more food than other farmers. | (Dixon et al., 2022) | 3.64 (0.69) | 0.777 | 0.81 | 0.596 |

***Note:*** CR refers to composite reliability; AVE refers to values of average variance extracted.

**Table S2.** Perceived benefits and risks related to agroforestry

**(a)** Perceived benefits

| **No.** | **Perceived benefits of agroofrestry** | **Mean** | ***SD*** |
| --- | --- | --- | --- |
| 1 | Agroforestry helps farmers increase their income via product diversification, such as growing fruits, nuts and timber. | 3.73 | 0.797 |
| 2 | Agroforestry improves animal health and welfare. | 3.72 | 0.815 |
| 3 | Agroforestry creates more habitats for wildlife. | 3.96 | 0.821 |
| 4 | Implementing agroforestry protects soil on farmlands. | 3.83 | 0.827 |
| 5 | Agroforestry helps control floods on farmlands. | 3.84 | 0.85 |
| 6 | Agroforestry helps reduce the impact of extreme weather, such as drought, on farming. | 3.72 | 0.865 |
| 7 | Agroforestry helps capture and store greenhouse gases. | 3.87 | 0.829 |
| 8 | Agroforestry increases crop and livestock productivity by improving farmland conditions. | 3.7 | 0.85 |
| 9 | Agroforestry will improve the appearance of landscapes. | 3.82 | 0.84 |

**(b)** Perceived risks

| **No.** | **Perceived risks of agroforestry** | **Mean** | ***SD*** |
| --- | --- | --- | --- |
| 1 | Agroforestry will lead to lower productivity on farms | 2.85 | 0.979 |
| 2 | Agroforestry will negatively affect the appearance of landscapes. | 2.62 | 1.056 |
| 3 | Agroforestry may involve planting non-native UK trees, potentially negatively affecting native plants, and animals | 3.13 | 0.954 |
| 4 | The presence of more trees on farmlands may potentially increase crime and reduce safety. | 2.63 | 1.1 |
| 5 | Agroforestry requires higher input in farmland management, such as more labour, mechanisation and knowledge. | 3.4 | 0.894 |
| 6 | Agroforestry makes farmland management and decisions about future land use more complicated | 3.19 | 0.93 |
| 7 | It takes a long time to grow the trees and see any benefit from them. | 3.56 | 0.942 |
| 8 | Agroforestry is a contested issue and can lead to conflict among different stakeholders | 3.26 | 0.882 |

**Table S3.** Reliability and validity tests of measurement and structural models

**(a)** Discriminant validity of measurement models

|  | **AtTC** | **PRtTRE** | **PfMCRL** | **PIoEC** | **PIoFP** | **Affect** | **BP** | **RP** | **AtA** |
| --- | --- | --- | --- | --- | --- | --- | --- | --- | --- |
| **AtTC_1** | **0.879** | 0.118 | 0.145 | 0.349 | 0.114 | 0.15 | 0.202 | -0.161 | 0.194 |
| **AtTC_2** | **0.9** | 0.147 | 0.18 | 0.368 | 0.138 | 0.173 | 0.227 | -0.134 | 0.206 |
| **AtTC_3** | **0.885** | 0.133 | 0.139 | 0.339 | 0.098 | 0.158 | 0.205 | -0.168 | 0.191 |
| **AtTC_4** | **0.785** | 0.174 | 0.189 | 0.304 | 0.165 | 0.18 | 0.243 | -0.106 | 0.218 |
| **PRtTRE_1** | 0.085 | **0.772** | 0.066 | 0.168 | 0.048 | 0.102 | 0.162 | 0.072 | 0.177 |
| **PRtTRE_2** | 0.15 | **0.777** | 0.142 | 0.191 | 0.035 | 0.093 | 0.169 | 0.05 | 0.121 |
| **PRtTRE_3** | 0.161 | **0.885** | 0.024 | 0.346 | 0.039 | 0.159 | 0.279 | -0.127 | 0.254 |
| **PfMCRL_1** | 0.169 | 0.06 | **0.881** | 0.096 | 0.187 | 0.102 | 0.118 | 0.089 | 0.084 |
| **PfMCRL_2** | 0.161 | 0.05 | **0.877** | 0.092 | 0.198 | 0.071 | 0.09 | 0.091 | 0.063 |
| **PfMCRL_3** | 0.157 | 0.097 | **0.799** | 0.119 | 0.196 | 0.072 | 0.115 | 0.124 | 0.07 |
| **PIoEC_1** | 0.301 | 0.234 | 0.095 | **0.778** | 0.332 | 0.278 | 0.42 | -0.251 | 0.42 |
| **PIoEC_2** | 0.319 | 0.206 | 0.071 | **0.803** | 0.261 | 0.253 | 0.363 | -0.253 | 0.357 |
| **PIoEC_3** | 0.29 | 0.227 | 0.076 | **0.782** | 0.302 | 0.274 | 0.419 | -0.216 | 0.394 |
| **PIoEC_4** | 0.265 | 0.259 | 0.054 | **0.793** | 0.231 | 0.283 | 0.397 | -0.235 | 0.377 |
| **PIoEC_5** | 0.345 | 0.263 | 0.113 | **0.777** | 0.218 | 0.254 | 0.377 | -0.248 | 0.369 |
| **PIoEC_6** | 0.316 | 0.261 | 0.114 | **0.75** | 0.174 | 0.212 | 0.329 | -0.247 | 0.311 |
| **PIoEC_7** | 0.316 | 0.242 | 0.138 | **0.765** | 0.183 | 0.238 | 0.347 | -0.251 | 0.312 |
| **PIoFP_1** | 0.156 | 0.068 | 0.107 | 0.29 | **0.829** | 0.258 | 0.246 | -0.009 | 0.27 |
| **PIoFP_2** | 0.069 | 0.07 | 0.168 | 0.237 | **0.812** | 0.166 | 0.176 | 0.055 | 0.196 |
| **PIoFP_3** | 0.15 | -0.012 | 0.248 | 0.245 | **0.757** | 0.154 | 0.179 | 0.06 | 0.194 |
| **PIoFP_4** | 0.064 | 0.013 | 0.217 | 0.183 | **0.682** | 0.15 | 0.111 | 0.171 | 0.152 |
| **Affect** | 0.192 | 0.153 | 0.097 | 0.33 | 0.244 | **1** | 0.532 | -0.315 | 0.57 |
| **BP_1** | 0.185 | 0.257 | 0.139 | 0.342 | 0.247 | 0.419 | **0.793** | -0.217 | 0.613 |
| **BP_2** | 0.251 | 0.22 | 0.12 | 0.475 | 0.194 | 0.473 | **0.888** | -0.426 | 0.674 |
| **BP_3** | 0.227 | 0.237 | 0.09 | 0.433 | 0.194 | 0.483 | **0.908** | -0.425 | 0.695 |
| **BP_4** | 0.226 | 0.213 | 0.1 | 0.45 | 0.21 | 0.483 | **0.903** | -0.403 | 0.721 |
| **RP_1** | -0.143 | -0.029 | 0.131 | -0.296 | 0.098 | -0.249 | -0.311 | **0.849** | -0.316 |
| **RP_2** | -0.155 | -0.038 | 0.072 | -0.298 | 0.044 | -0.295 | -0.389 | **0.919** | -0.399 |
| **RP_3** | -0.134 | -0.022 | 0.109 | -0.244 | 0.058 | -0.302 | -0.423 | **0.917** | -0.415 |
| **RP_4** | -0.159 | -0.021 | 0.121 | -0.287 | 0.068 | -0.289 | -0.405 | **0.919** | -0.415 |
| **AtA_1** | 0.219 | 0.218 | 0.091 | 0.449 | 0.245 | 0.555 | 0.686 | -0.423 | **0.893** |
| **AtA_2** | 0.192 | 0.182 | 0.066 | 0.403 | 0.246 | 0.486 | 0.688 | -0.374 | **0.905** |
| **AtA_3** | 0.222 | 0.243 | 0.072 | 0.411 | 0.242 | 0.495 | 0.715 | -0.362 | **0.9** |

***Note:*** AtTC refers to attachment to the countryside; PRtTRE refers to perceived risk to the rural environment; PfMCRL refers to preference for maintaining the current rural landscape; PioEC refers to perceived importance of environmental conservation; PioFP refers to perceived importance of food productivity; Affect refers to affect evoked by agroforestry; BP refers to benefit perception of agroforestry; RP refers to risk perception of agroforestry; AtA refers to attitudes towards agroforestry.

**(b)** Fornell-Larcker Criterion

|  | **Affect** | **AtA** | **AtTC** | **BP** | **PIoEC** | **PIoFP** | **PRtTRE** | **PfMCRL** | **RP** |
| --- | --- | --- | --- | --- | --- | --- | --- | --- | --- |
| **Affect** | **1** |  |  |  |  |  |  |  |  |
| **AtA** | 0.57 | **0.899** |  |  |  |  |  |  |  |
| **AtTC** | 0.192 | 0.235 | **0.863** |  |  |  |  |  |  |
| **BP** | 0.532 | 0.774 | 0.255 | **0.874** |  |  |  |  |  |
| **PIoEC** | 0.33 | 0.469 | 0.395 | 0.488 | **0.778** |  |  |  |  |
| **PIoFP** | 0.244 | 0.272 | 0.15 | 0.24 | 0.316 | **0.772** |  |  |  |
| **PRtTRE** | 0.153 | 0.239 | 0.166 | 0.264 | 0.31 | 0.049 | **0.813** |  |  |
| **PfMCRL** | 0.097 | 0.085 | 0.191 | 0.127 | 0.12 | 0.227 | 0.081 | **0.853** |  |
| **RP** | -0.315 | -0.43 | -0.164 | -0.425 | -0.312 | 0.073 | -0.03 | 0.12 | **0.901** |

**(c)** Heterotrait-Monotrait Ratio

|  | **Affect** | **AtA** | **AtTC** | **BP** | **PIoEC** | **PIoFP** | **PRtTRE** | **PfMCRL** | **RP** |
| --- | --- | --- | --- | --- | --- | --- | --- | --- | --- |
| **Affect** |  |  |  |  |  |  |  |  |  |
| **AtA** | 0.607 |  |  |  |  |  |  |  |  |
| **AtTC** | 0.203 | 0.266 |  |  |  |  |  |  |  |
| **BP** | 0.562 | 0.871 | 0.285 |  |  |  |  |  |  |
| **PIoEC** | 0.348 | 0.526 | 0.444 | 0.542 |  |  |  |  |  |
| **PIoFP** | 0.267 | 0.317 | 0.17 | 0.278 | 0.366 |  |  |  |  |
| **PRtTRE** | 0.167 | 0.275 | 0.197 | 0.305 | 0.351 | 0.086 |  |  |  |
| **PfMCRL** | 0.106 | 0.1 | 0.223 | 0.15 | 0.142 | 0.301 | 0.12 |  |  |
| **RP** | 0.328 | 0.475 | 0.183 | 0.462 | 0.345 | 0.12 | 0.123 | 0.138 |  |

**(d)** Inner variance inflation factor (VIF) values

|  | **Affect** | **AtA** | **AtTC** | **BP** | **PIoEC** | **PIoFP** | **PRtTRE** | **PfMCRL** | **RP** |
| --- | --- | --- | --- | --- | --- | --- | --- | --- | --- |
| **Affect** |  | 1.462 |  | 1.161 |  |  |  |  | 1.161 |
| **AtA** |  |  |  |  |  |  |  |  |  |
| **AtTC** | 1.217 | 1.227 |  | 1.221 | 1 | 1 | 1 | 1 | 1.221 |
| **BP** |  | 1.848 |  |  |  |  |  |  |  |
| **PIoEC** | 1.392 | 1.666 |  | 1.457 |  |  |  |  | 1.457 |
| **PIoFP** | 1.162 | 1.262 |  | 1.189 |  |  |  |  | 1.189 |
| **PRtTRE** | 1.115 | 1.161 |  | 1.12 |  |  |  |  | 1.12 |
| **PfMCRL** | 1.086 | 1.12 |  | 1.086 |  |  |  |  | 1.086 |
| **RP** |  | 1.417 |  |  |  |  |  |  |  |

**(e)** Predictive power of the model

|  | **R-square** | **R-square adjusted** |
| --- | --- | --- |
| **Affect** | 0.138 | 0.136 |
| **AtA** | **0.654** | **0.652** |
| **BP** | 0.406 | 0.403 |
| **PIoEC** | 0.156 | 0.155 |
| **PIoFP** | 0.022 | 0.022 |
| **PRtTRE** | 0.028 | 0.027 |
| **PfMCRL** | 0.036 | 0.036 |
| **RP** | 0.225 | 0.221 |

**Table S4.** Effects of target constructs on attitudes towards agroforestry implementation. *, **, and *** indicate significance at *p* < 0.05, 0.01, and 0.001, respectively.

**(a)** Whole sample

| **Constructs** |  | **Direct effects** | **Indirect effects** | **Total effects** |  |
| --- | --- | --- | --- | --- | --- |
| Benefit perception of agroforestry | | 0.572*** | NA | 0.572*** |  |
| Risk perception of agroforestry | | -0.112*** | NA | -0.112*** | |
| Affect evoked by agroforestry | | 0.188*** | 0.261*** | 0.449*** |  |
| Perceived importance of food productivity | | 0.081*** | 0.063** | 0.144*** |  |
| Perceived importance of environmental conservation | | 0.059** | 0.311*** | 0.370*** |  |
| Perceived threat to the rural environment | | 0.036* | 0.074*** | 0.110*** |  |
| Preference for maintaining current rural landscape | | -0.020 | 0.010 | -0.011 |  |
| Attachment to the countryside | | -0.003 | 0.238*** | 0.235*** |  |

**(b)** Segment 1: cautious conservation-oriented citizens

| **Constructs** |  | **Direct effects** | **Indirect effects** | **Total effects** |  |
| --- | --- | --- | --- | --- | --- |
| Benefit perception of agroforestry | | 0.388*** | NA | 0.388*** |  |
| Risk perception of agroforestry | | -0.251*** | NA | -0.251*** | |
| Affect evoked by agroforestry | | 0.200*** | 0.449*** | 0.648*** |  |
| Perceived importance of food productivity | | -0.001 | 0.03 | 0.029 |  |
| Perceived importance of environmental conservation | | 0.149*** | 0.308*** | 0.457*** |  |
| Perceived threat to the rural environment | | -0.017 | 0.057 | 0.039 |  |
| Preference for maintaining current rural landscape | | 0.011 | -0.01 | 0.001 |  |
| Attachment to the countryside | | -0.029 | 0.187*** | 0.159*** |  |

**(c)** Segment 2: *citizens sensitive to threats to the rural environment*

| **Constructs** |  | **Direct effects** | **Indirect effects** | **Total effects** |  |
| --- | --- | --- | --- | --- | --- |
| Benefit perception of agroforestry | | 0.533*** | NA | 0.533*** |  |
| Risk perception of agroforestry | | -0.131*** | NA | -0.131*** | |
| Affect evoked by agroforestry | | 0.197*** | 0.391*** | 0.588*** |  |
| Perceived importance of food productivity | | 0.05 | 0.007 | 0.057 |  |
| Perceived importance of environmental conservation | | 0.023 | 0.267*** | 0.289*** |  |
| Perceived threat to the rural environment | | 0.111*** | 0.250*** | 0.360*** |  |
| Preference for maintaining current rural landscape | | -0.087** | -0.007 | -0.094 |  |
| Attachment to the countryside | | 0.008 | 0.164*** | 0.172*** |  |

**(d)** Segment 3: *countryside-engaged eco-productive citizens*

| **Constructs** |  | **Direct effects** | **Indirect effects** | **Total effects** |  |
| --- | --- | --- | --- | --- | --- |
| Benefit perception of agroforestry | | 0.597*** | NA | 0.597*** |  |
| Risk perception of agroforestry | | -0.063* | NA | -0.063* | |
| Affect evoked by agroforestry | | 0.099*** | 0.007 | 0.105** |  |
| Perceived importance of food productivity | | 0.206*** | 0.177*** | 0.383*** |  |
| Perceived importance of environmental conservation | | 0.049 | 0.323*** | 0.372*** |  |
| Perceived threat to the rural environment | | 0.001 | -0.065** | -0.063 |  |
| Preference for maintaining current rural landscape | | 0.009 | 0.02 | 0.029 |  |
| Attachment to the countryside | | -0.021 | 0.392*** | 0.372*** |  |

**Table S5.** Parameters for determining the number of segments using FIMIX-PLS

|  | **S1** | **S2** | **S3** | **S4** | **S5** |
| --- | --- | --- | --- | --- | --- |
| **AIC (Akaike's information criterion)** | 30948.438 | 29274.83 | 28965.792 | 28813.868 | 28529.116 |
| **AIC3 (modified AIC with Factor 3)** | 30985.438 | 29349.83 | 29078.792 | 28964.868 | 28718.116 |
| **AIC4 (modified AIC with Factor 4)** | 31022.438 | 29424.83 | 29191.792 | 29115.868 | 28907.116 |
| **BIC (Bayesian information criterion)** | 31145.249 | 29673.77 | 29566.861 | 29617.067 | 29534.445 |
| **CAIC (consistent AIC)** | 31182.249 | 29748.77 | 29679.861 | 29768.067 | 29723.445 |
| **HQ (Hannan-Quinn criterion)** | 31021.736 | 29423.405 | 29189.645 | 29112.999 | 28903.526 |
| **MDL5 (minimum description length with factor 5)** | 32228.491 | 31869.531 | 32875.141 | 34037.865 | 35067.762 |
| **LnL (LogLikelihood)** | -15437.219 | -14562.415 | -14369.896 | -14255.934 | -14075.558 |
| **EN (normed entropy statistic)** | 0 | 0.946 | 0.688 | 0.643 | 0.676 |
| **NFI (non-fuzzy index)** | 0 | 0.962 | 0.679 | 0.618 | 0.627 |
| **NEC (normalized entropy criterion)** | 0 | 81.088 | 470.785 | 539.356 | 488.374 |

**Table S6.** Reliability and validity tests of measurement and structural models

**Group 1:**

**(a)** Internal reliability of construct measurement

|  | **Cronbach's alpha** | **Composite reliability (rho_a)** | **Composite reliability (rho_c)** | **Average variance extracted (AVE)** |
| --- | --- | --- | --- | --- |
| **AtA** | 0.853 | 0.854 | 0.911 | 0.772 |
| **AtTC** | 0.877 | 0.88 | 0.916 | 0.731 |
| **BP** | 0.879 | 0.881 | 0.917 | 0.734 |
| **PIoEC** | 0.883 | 0.89 | 0.909 | 0.588 |
| **PIoFP** | 0.802 | 0.808 | 0.871 | 0.627 |
| **PRtTRE** | 0.783 | 0.791 | 0.873 | 0.696 |
| **PfMCRL** | 0.793 | 0.801 | 0.879 | 0.707 |
| **RP** | 0.925 | 0.927 | 0.947 | 0.816 |

**(b)** Discriminant validity of measurement models

|  | **Affect** | **AtA** | **AtTC** | **BP** | **PIoEC** | **PIoFP** | **PRtTRE** | **PfMCRL** | **RP** |
| --- | --- | --- | --- | --- | --- | --- | --- | --- | --- |
| **AtTC_1** | 0.212 | 0.25 | **0.863** | 0.262 | 0.381 | 0.227 | 0.114 | 0.193 | -0.143 |
| **AtTC_2** | 0.225 | 0.282 | **0.889** | 0.267 | 0.4 | 0.251 | 0.132 | 0.225 | -0.107 |
| **AtTC_3** | 0.186 | 0.223 | **0.874** | 0.245 | 0.308 | 0.204 | 0.117 | 0.23 | -0.125 |
| **AtTC_4** | 0.239 | 0.244 | **0.792** | 0.267 | 0.29 | 0.234 | 0.133 | 0.258 | -0.065 |
| **PRtTRE_1** | 0.109 | 0.197 | 0.104 | 0.151 | 0.184 | 0.122 | **0.841** | 0.137 | 0.171 |
| **PRtTRE_2** | 0.116 | 0.116 | 0.128 | 0.14 | 0.162 | 0.065 | **0.819** | 0.137 | 0.133 |
| **PRtTRE_3** | 0.125 | 0.208 | 0.133 | 0.251 | 0.318 | 0.066 | **0.842** | 0.041 | -0.028 |
| **PfMCRL_1** | 0.162 | 0.172 | 0.259 | 0.219 | 0.16 | 0.256 | 0.108 | **0.873** | 0.086 |
| **PfMCRL_2** | 0.151 | 0.153 | 0.211 | 0.183 | 0.17 | 0.226 | 0.11 | **0.849** | 0.084 |
| **PfMCRL_3** | 0.123 | 0.135 | 0.191 | 0.195 | 0.171 | 0.241 | 0.089 | **0.8** | 0.149 |
| **Affect** | **1** | 0.6 | 0.253 | 0.556 | 0.316 | 0.359 | 0.14 | 0.174 | -0.246 |
| **BP_1** | 0.448 | 0.618 | 0.272 | **0.797** | 0.431 | 0.414 | 0.202 | 0.242 | -0.138 |
| **BP_2** | 0.481 | 0.643 | 0.267 | **0.86** | 0.543 | 0.344 | 0.168 | 0.193 | -0.29 |
| **BP_3** | 0.472 | 0.665 | 0.239 | **0.885** | 0.474 | 0.325 | 0.207 | 0.182 | -0.321 |
| **BP_4** | 0.503 | 0.696 | 0.269 | **0.883** | 0.519 | 0.325 | 0.184 | 0.2 | -0.299 |
| **RP_1** | -0.192 | -0.201 | -0.084 | -0.236 | -0.225 | 0.039 | 0.077 | 0.161 | **0.863** |
| **RP_2** | -0.249 | -0.249 | -0.136 | -0.304 | -0.25 | 0.002 | 0.093 | 0.063 | **0.922** |
| **RP_3** | -0.222 | -0.243 | -0.113 | -0.283 | -0.168 | 0.034 | 0.114 | 0.088 | **0.91** |
| **RP_4** | -0.223 | -0.278 | -0.13 | -0.289 | -0.213 | 0.034 | 0.091 | 0.138 | **0.917** |
| **AtA_1** | 0.59 | **0.876** | 0.281 | 0.67 | 0.507 | 0.391 | 0.219 | 0.164 | -0.27 |
| **AtA_2** | 0.479 | **0.872** | 0.226 | 0.663 | 0.4 | 0.352 | 0.144 | 0.157 | -0.235 |
| **AtA_3** | 0.509 | **0.888** | 0.264 | 0.686 | 0.467 | 0.385 | 0.194 | 0.162 | -0.205 |
| **PIoFP_1** | 0.353 | 0.384 | 0.237 | 0.367 | 0.37 | **0.808** | 0.085 | 0.179 | -0.049 |
| **PIoFP_2** | 0.226 | 0.304 | 0.188 | 0.295 | 0.359 | **0.811** | 0.118 | 0.211 | 0.001 |
| **PIoFP_3** | 0.255 | 0.339 | 0.213 | 0.334 | 0.369 | **0.799** | 0.032 | 0.301 | 0.031 |
| **PIoFP_4** | 0.285 | 0.319 | 0.207 | 0.29 | 0.305 | **0.748** | 0.092 | 0.225 | 0.127 |
| **PIoEC_1** | 0.296 | 0.511 | 0.29 | 0.513 | **0.799** | 0.422 | 0.224 | 0.157 | -0.222 |
| **PIoEC_2** | 0.226 | 0.394 | 0.292 | 0.433 | **0.812** | 0.375 | 0.206 | 0.125 | -0.162 |
| **PIoEC_3** | 0.288 | 0.455 | 0.314 | 0.489 | **0.801** | 0.418 | 0.241 | 0.15 | -0.142 |
| **PIoEC_4** | 0.303 | 0.434 | 0.292 | 0.465 | **0.804** | 0.36 | 0.279 | 0.092 | -0.178 |
| **PIoEC_5** | 0.202 | 0.371 | 0.362 | 0.45 | **0.751** | 0.288 | 0.185 | 0.186 | -0.185 |
| **PIoEC_6** | 0.184 | 0.321 | 0.328 | 0.362 | **0.702** | 0.24 | 0.165 | 0.176 | -0.204 |
| **PIoEC_7** | 0.16 | 0.266 | 0.317 | 0.337 | **0.686** | 0.234 | 0.136 | 0.196 | -0.188 |

**(c)** Fornell-Larcker Criterion

|  | **Affect** | **AtA** | **AtTC** | **BP** | **PIoEC** | **PIoFP** | **PRtTRE** | **PfMCRL** | **RP** |
| --- | --- | --- | --- | --- | --- | --- | --- | --- | --- |
| **Affect** | 1 |  |  |  |  |  |  |  |  |
| **AtA** | 0.6 | 0.879 |  |  |  |  |  |  |  |
| **AtTC** | 0.253 | 0.294 | 0.855 |  |  |  |  |  |  |
| **BP** | 0.556 | 0.766 | 0.305 | 0.857 |  |  |  |  |  |
| **PIoEC** | 0.316 | 0.523 | 0.406 | 0.575 | 0.767 |  |  |  |  |
| **PIoFP** | 0.359 | 0.429 | 0.269 | 0.409 | 0.444 | 0.792 |  |  |  |
| **PRtTRE** | 0.14 | 0.213 | 0.145 | 0.221 | 0.272 | 0.102 | 0.834 |  |  |
| **PfMCRL** | 0.174 | 0.183 | 0.264 | 0.237 | 0.198 | 0.287 | 0.122 | 0.841 |  |
| **RP** | -0.246 | -0.27 | -0.129 | -0.308 | -0.237 | 0.03 | 0.103 | 0.124 | 0.903 |

**(d)** Heterotrait-Monotrait Ratio

|  | **Affect** | **AtA** | **AtTC** | **BP** | **PIoEC** | **PIoFP** | **PRtTRE** | **PfMCRL** | **RP** |
| --- | --- | --- | --- | --- | --- | --- | --- | --- | --- |
| **Affect** |  |  |  |  |  |  |  |  |  |
| **AtA** | 0.648 |  |  |  |  |  |  |  |  |
| **AtTC** | 0.27 | 0.337 |  |  |  |  |  |  |  |
| **BP** | 0.593 | 0.884 | 0.348 |  |  |  |  |  |  |
| **PIoEC** | 0.329 | 0.59 | 0.463 | 0.645 |  |  |  |  |  |
| **PIoFP** | 0.394 | 0.513 | 0.317 | 0.485 | 0.517 |  |  |  |  |
| **PRtTRE** | 0.158 | 0.253 | 0.175 | 0.262 | 0.314 | 0.129 |  |  |  |
| **PfMCRL** | 0.194 | 0.222 | 0.315 | 0.285 | 0.242 | 0.362 | 0.16 |  |  |
| **RP** | 0.255 | 0.302 | 0.142 | 0.339 | 0.263 | 0.08 | 0.156 | 0.148 |  |

**(e)** Inner variance inflation factor (VIF) values

|  | **Affect** | **AtA** | **AtTC** | **BP** | **PIoEC** | **PIoFP** | **PRtTRE** | **PfMCRL** | **RP** |
| --- | --- | --- | --- | --- | --- | --- | --- | --- | --- |
| **Affect** |  | 1.547 |  | 1.214 |  |  |  |  | 1.214 |
| **AtA** |  |  |  |  |  |  |  |  |  |
| **AtTC** | 1.256 | 1.277 |  | 1.272 | 1 | 1 | 1 | 1 | 1.272 |
| **BP** |  | 2.084 |  |  |  |  |  |  |  |
| **PIoEC** | 1.477 | 1.863 |  | 1.499 |  |  |  |  | 1.499 |
| **PIoFP** | 1.321 | 1.477 |  | 1.398 |  |  |  |  | 1.398 |
| **PRtTRE** | 1.088 | 1.15 |  | 1.092 |  |  |  |  | 1.092 |
| **PfMCRL** | 1.143 | 1.196 |  | 1.145 |  |  |  |  | 1.145 |
| **RP** |  | 1.295 |  |  |  |  |  |  |  |

**(f)** Predictive power of the model

|  | **R-square** | **R-square adjusted** |
| --- | --- | --- |
| **Affect** | 0.176 | 0.168 |
| **AtA** | 0.647 | 0.641 |
| **BP** | 0.498 | 0.492 |
| **PIoEC** | 0.165 | 0.163 |
| **PIoFP** | 0.072 | 0.07 |
| **PRtTRE** | 0.021 | 0.019 |
| **PfMCRL** | 0.07 | 0.068 |
| **RP** | 0.193 | 0.183 |

**Group 2:**

**(a)** Internal reliability of construct measurement

|  | **Cronbach's alpha** | **Composite reliability (rho_a)** | **Composite reliability (rho_c)** | **Average variance extracted (AVE)** |
| --- | --- | --- | --- | --- |
| **AtA** | 0.893 | 0.893 | 0.933 | 0.823 |
| **AtTC** | 0.892 | 0.894 | 0.926 | 0.757 |
| **BP** | 0.902 | 0.91 | 0.932 | 0.776 |
| **PIoEC** | 0.893 | 0.894 | 0.916 | 0.61 |
| **PIoFP** | 0.754 | 0.856 | 0.834 | 0.562 |
| **PRtTRE** | 0.718 | 0.821 | 0.834 | 0.628 |
| **PfMCRL** | 0.807 | 0.808 | 0.886 | 0.722 |
| **RP** | 0.916 | 0.92 | 0.941 | 0.799 |

**(b)** Discriminant validity of measurement models

|  | **Affect** | **AtA** | **AtTC** | **BP** | **PIoEC** | **PIoFP** | **PRtTRE** | **PfMCRL** | **RP** |
| --- | --- | --- | --- | --- | --- | --- | --- | --- | --- |
| **AtTC_1** | 0.167 | 0.198 | **0.881** | 0.222 | 0.345 | 0.112 | 0.184 | 0.159 | -0.177 |
| **AtTC_2** | 0.199 | 0.213 | **0.904** | 0.27 | 0.381 | 0.114 | 0.217 | 0.2 | -0.178 |
| **AtTC_3** | 0.212 | 0.238 | **0.909** | 0.277 | 0.377 | 0.098 | 0.213 | 0.153 | -0.215 |
| **AtTC_4** | 0.196 | 0.248 | **0.781** | 0.279 | 0.326 | 0.167 | 0.233 | 0.172 | -0.14 |
| **PRtTRE_1** | 0.113 | 0.165 | 0.161 | 0.155 | 0.16 | -0.027 | **0.738** | 0.079 | 0.025 |
| **PRtTRE_2** | 0.067 | 0.102 | 0.216 | 0.183 | 0.213 | 0.056 | **0.746** | 0.157 | 0.03 |
| **PRtTRE_3** | 0.203 | 0.288 | 0.208 | 0.307 | 0.349 | 0.054 | **0.884** | 0.052 | -0.149 |
| **PfMCRL_1** | 0.04 | 0.061 | 0.17 | 0.103 | 0.122 | 0.085 | 0.088 | **0.874** | 0.048 |
| **PfMCRL_2** | -0.005 | 0.03 | 0.168 | 0.072 | 0.093 | 0.132 | 0.056 | **0.876** | 0.056 |
| **PfMCRL_3** | 0.045 | 0.073 | 0.162 | 0.115 | 0.111 | 0.152 | 0.12 | **0.798** | 0.068 |
| **Affect** | **1** | 0.56 | 0.224 | 0.533 | 0.373 | 0.2 | 0.178 | 0.033 | -0.354 |
| **BP_1** | 0.393 | 0.603 | 0.2 | **0.782** | 0.344 | 0.183 | 0.287 | 0.11 | -0.25 |
| **BP_2** | 0.478 | 0.682 | 0.312 | **0.906** | 0.479 | 0.165 | 0.266 | 0.126 | -0.502 |
| **BP_3** | 0.504 | 0.702 | 0.291 | **0.916** | 0.459 | 0.16 | 0.258 | 0.093 | -0.501 |
| **BP_4** | 0.496 | 0.743 | 0.256 | **0.911** | 0.455 | 0.207 | 0.22 | 0.082 | -0.467 |
| **RP_1** | -0.291 | -0.369 | -0.188 | -0.364 | -0.335 | 0.062 | -0.062 | 0.056 | **0.832** |
| **RP_2** | -0.319 | -0.471 | -0.175 | -0.433 | -0.309 | 0.003 | -0.059 | 0.036 | **0.91** |
| **RP_3** | -0.336 | -0.505 | -0.168 | -0.498 | -0.291 | 0.011 | -0.069 | 0.085 | **0.915** |
| **RP_4** | -0.317 | -0.486 | -0.201 | -0.467 | -0.328 | 0.026 | -0.058 | 0.064 | **0.915** |
| **AtA_1** | 0.54 | **0.898** | 0.235 | 0.692 | 0.433 | 0.218 | 0.227 | 0.082 | -0.511 |
| **AtA_2** | 0.489 | **0.915** | 0.204 | 0.696 | 0.408 | 0.244 | 0.186 | 0.034 | -0.455 |
| **AtA_3** | 0.494 | **0.909** | 0.265 | 0.727 | 0.4 | 0.221 | 0.282 | 0.063 | -0.435 |
| **PIoFP_1** | 0.24 | 0.258 | 0.144 | 0.222 | 0.299 | **0.862** | 0.08 | 0.057 | -0.035 |
| **PIoFP_2** | 0.124 | 0.161 | 0.034 | 0.125 | 0.182 | **0.797** | 0.023 | 0.138 | 0.05 |
| **PIoFP_3** | 0.099 | 0.173 | 0.16 | 0.144 | 0.204 | **0.739** | -0.021 | 0.161 | 0.028 |
| **PIoFP_4** | 0.059 | 0.099 | 0.025 | 0.033 | 0.145 | **0.568** | 0.004 | 0.156 | 0.149 |
| **PIoEC_1** | 0.277 | 0.374 | 0.322 | 0.422 | **0.761** | 0.313 | 0.255 | 0.101 | -0.266 |
| **PIoEC_2** | 0.294 | 0.356 | 0.336 | 0.378 | **0.773** | 0.246 | 0.209 | 0.084 | -0.308 |
| **PIoEC_3** | 0.294 | 0.359 | 0.283 | 0.423 | **0.789** | 0.269 | 0.236 | 0.076 | -0.276 |
| **PIoEC_4** | 0.297 | 0.362 | 0.262 | 0.394 | **0.785** | 0.195 | 0.251 | 0.061 | -0.261 |
| **PIoEC_5** | 0.297 | 0.365 | 0.38 | 0.365 | **0.771** | 0.215 | 0.258 | 0.09 | -0.269 |
| **PIoEC_6** | 0.258 | 0.316 | 0.335 | 0.337 | **0.776** | 0.157 | 0.28 | 0.123 | -0.283 |
| **PIoEC_7** | 0.319 | 0.359 | 0.331 | 0.388 | **0.812** | 0.21 | 0.295 | 0.171 | -0.261 |

**(c)** Fornell-Larcker Criterion

|  | **Affect** | **AtA** | **AtTC** | **BP** | **PIoEC** | **PIoFP** | **PRtTRE** | **PfMCRL** | **RP** |
| --- | --- | --- | --- | --- | --- | --- | --- | --- | --- |
| **Affect** | 1 |  |  |  |  |  |  |  |  |
| **AtA** | 0.56 | 0.907 |  |  |  |  |  |  |  |
| **AtTC** | 0.224 | 0.259 | 0.87 |  |  |  |  |  |  |
| **BP** | 0.533 | 0.777 | 0.303 | 0.881 |  |  |  |  |  |
| **PIoEC** | 0.373 | 0.456 | 0.412 | 0.496 | 0.781 |  |  |  |  |
| **PIoFP** | 0.2 | 0.251 | 0.141 | 0.202 | 0.295 | 0.75 |  |  |  |
| **PRtTRE** | 0.178 | 0.256 | 0.244 | 0.29 | 0.326 | 0.041 | 0.792 |  |  |
| **PfMCRL** | 0.033 | 0.066 | 0.197 | 0.116 | 0.129 | 0.145 | 0.106 | 0.85 |  |
| **RP** | -0.354 | -0.515 | -0.205 | -0.495 | -0.352 | 0.028 | -0.07 | 0.068 | 0.894 |

**(d)** Heterotrait-Monotrait Ratio

|  | **Affect** | **AtA** | **AtTC** | **BP** | **PIoEC** | **PIoFP** | **PRtTRE** | **PfMCRL** | **RP** |
| --- | --- | --- | --- | --- | --- | --- | --- | --- | --- |
| **Affect** |  |  |  |  |  |  |  |  |  |
| **AtA** | 0.592 |  |  |  |  |  |  |  |  |
| **AtTC** | 0.236 | 0.289 |  |  |  |  |  |  |  |
| **BP** | 0.56 | 0.865 | 0.334 |  |  |  |  |  |  |
| **PIoEC** | 0.394 | 0.51 | 0.46 | 0.549 |  |  |  |  |  |
| **PIoFP** | 0.198 | 0.278 | 0.158 | 0.214 | 0.332 |  |  |  |  |
| **PRtTRE** | 0.189 | 0.288 | 0.304 | 0.338 | 0.377 | 0.093 |  |  |  |
| **PfMCRL** | 0.039 | 0.076 | 0.231 | 0.135 | 0.151 | 0.215 | 0.155 |  |  |
| **RP** | 0.369 | 0.567 | 0.227 | 0.535 | 0.391 | 0.107 | 0.107 | 0.078 |  |

**(e)** Inner variance inflation factor (VIF) values

|  | **Affect** | **AtA** | **AtTC** | **BP** | **PIoEC** | **PIoFP** | **PRtTRE** | **PfMCRL** | **RP** |
| --- | --- | --- | --- | --- | --- | --- | --- | --- | --- |
| **Affect** |  | 1.463 |  | 1.188 |  |  |  |  | 1.188 |
| **AtA** |  |  |  |  |  |  |  |  |  |
| **AtTC** | 1.253 | 1.272 |  | 1.261 | 1 | 1 | 1 | 1 | 1.261 |
| **BP** |  | 1.929 |  |  |  |  |  |  |  |
| **PIoEC** | 1.389 | 1.675 |  | 1.491 |  |  |  |  | 1.491 |
| **PIoFP** | 1.115 | 1.175 |  | 1.128 |  |  |  |  | 1.128 |
| **PRtTRE** | 1.146 | 1.194 |  | 1.15 |  |  |  |  | 1.15 |
| **PfMCRL** | 1.06 | 1.084 |  | 1.062 |  |  |  |  | 1.062 |
| **RP** |  | 1.482 |  |  |  |  |  |  |  |

**(f)** Predictive power of the model

|  | **R-square** | **R-square adjusted** |
| --- | --- | --- |
| **Affect** | 0.158 | 0.152 |
| **AtA** | **0.662** | **0.659** |
| **BP** | 0.405 | 0.4 |
| **PIoEC** | 0.17 | 0.168 |
| **PIoFP** | 0.02 | 0.019 |
| **PRtTRE** | 0.06 | 0.058 |
| **PfMCRL** | 0.039 | 0.037 |
| **RP** | 0.226 | 0.22 |

**Group 3:**

**(a)** Internal reliability of construct measurement

|  | **Cronbach's alpha** | **Composite reliability (rho_a)** | **Composite reliability (rho_c)** | **Average variance extracted (AVE)** |
| --- | --- | --- | --- | --- |
| **AtA** | 0.901 | 0.901 | 0.938 | 0.835 |
| **AtTC** | 0.876 | 0.878 | 0.915 | 0.731 |
| **BP** | 0.911 | 0.915 | 0.938 | 0.79 |
| **PIoEC** | 0.906 | 0.907 | 0.926 | 0.64 |
| **PIoFP** | 0.753 | 0.811 | 0.836 | 0.561 |
| **PRtTRE** | 0.788 | 0.914 | 0.87 | 0.691 |
| **PfMCRL** | 0.842 | 0.842 | 0.906 | 0.763 |
| **RP** | 0.931 | 0.932 | 0.951 | 0.829 |

**(b)** Discriminant validity of measurement models

|  | **Affect** | **AtA** | **AtTC** | **BP** | **PIoEC** | **PIoFP** | **PRtTRE** | **PfMCRL** | **RP** |
| --- | --- | --- | --- | --- | --- | --- | --- | --- | --- |
| **AtTC_1** | 0.03 | 0.126 | **0.906** | 0.087 | 0.28 | 0 | -0.01 | 0.096 | -0.117 |
| **AtTC_2** | 0.048 | 0.089 | **0.903** | 0.083 | 0.256 | 0.079 | 0.007 | 0.128 | -0.038 |
| **AtTC_3** | 0.04 | 0.087 | **0.84** | 0.015 | 0.294 | 0.004 | -0.02 | 0.04 | -0.087 |
| **AtTC_4** | 0.058 | 0.127 | **0.764** | 0.136 | 0.257 | 0.083 | 0.103 | 0.181 | -0.069 |
| **PRtTRE_1** | 0.04 | 0.152 | -0.062 | 0.178 | 0.189 | -0.004 | **0.781** | -0.084 | -0.039 |
| **PRtTRE_2** | 0.128 | 0.19 | 0.015 | 0.202 | 0.198 | -0.033 | **0.797** | 0.144 | -0.049 |
| **PRtTRE_3** | 0.145 | 0.282 | 0.069 | 0.29 | 0.39 | -0.013 | **0.91** | -0.031 | -0.235 |
| **PfMCRL_1** | 0.121 | -0.03 | 0.08 | -0.036 | -0.048 | 0.279 | -0.035 | **0.897** | 0.152 |
| **PfMCRL_2** | 0.096 | -0.019 | 0.128 | -0.03 | -0.012 | 0.316 | -0.027 | **0.92** | 0.153 |
| **PfMCRL_3** | 0.028 | -0.061 | 0.142 | -0.031 | 0.06 | 0.196 | 0.084 | **0.799** | 0.177 |
| **Affect** | **1** | 0.533 | 0.052 | 0.476 | 0.28 | 0.101 | 0.137 | 0.092 | -0.42 |
| **BP_1** | 0.413 | 0.62 | 0.072 | **0.814** | 0.211 | 0.008 | 0.305 | 0.012 | -0.369 |
| **BP_2** | 0.437 | 0.705 | 0.114 | **0.894** | 0.359 | -0.066 | 0.211 | -0.023 | -0.554 |
| **BP_3** | 0.442 | 0.731 | 0.06 | **0.931** | 0.297 | -0.019 | 0.24 | -0.082 | -0.499 |
| **BP_4** | 0.4 | 0.705 | 0.104 | **0.912** | 0.324 | -0.033 | 0.253 | -0.032 | -0.498 |
| **RP_1** | -0.335 | -0.491 | -0.084 | -0.405 | -0.348 | 0.229 | -0.113 | 0.2 | **0.853** |
| **RP_2** | -0.367 | -0.545 | -0.11 | -0.483 | -0.357 | 0.173 | -0.207 | 0.128 | **0.932** |
| **RP_3** | -0.417 | -0.564 | -0.065 | -0.553 | -0.275 | 0.175 | -0.143 | 0.163 | **0.928** |
| **RP_4** | -0.406 | -0.562 | -0.074 | -0.533 | -0.333 | 0.192 | -0.121 | 0.182 | **0.926** |
| **AtA_1** | 0.519 | **0.914** | 0.12 | 0.699 | 0.407 | -0.023 | 0.214 | -0.023 | -0.566 |
| **AtA_2** | 0.48 | **0.932** | 0.151 | 0.701 | 0.418 | -0.007 | 0.259 | -0.023 | -0.507 |
| **AtA_3** | 0.461 | **0.895** | 0.081 | 0.732 | 0.363 | -0.039 | 0.257 | -0.071 | -0.555 |
| **PIoFP_1** | 0.069 | 0.044 | 0.08 | 0.034 | 0.125 | **0.681** | 0.029 | 0.073 | 0.092 |
| **PIoFP_2** | 0.119 | 0.056 | -0.001 | 0.052 | 0.172 | **0.745** | 0.111 | 0.136 | 0.119 |
| **PIoFP_3** | 0.063 | -0.039 | 0.111 | -0.042 | 0.154 | **0.736** | -0.046 | 0.313 | 0.127 |
| **PIoFP_4** | 0.067 | -0.077 | -0.007 | -0.082 | 0.09 | **0.828** | -0.085 | 0.3 | 0.238 |
| **PIoEC_1** | 0.248 | 0.369 | 0.292 | 0.239 | **0.788** | 0.196 | 0.194 | -0.013 | -0.297 |
| **PIoEC_2** | 0.225 | 0.321 | 0.303 | 0.218 | **0.865** | 0.138 | 0.188 | -0.015 | -0.29 |
| **PIoEC_3** | 0.199 | 0.375 | 0.276 | 0.285 | **0.735** | 0.163 | 0.171 | -0.039 | -0.235 |
| **PIoEC_4** | 0.226 | 0.334 | 0.222 | 0.298 | **0.793** | 0.097 | 0.234 | 0 | -0.279 |
| **PIoEC_5** | 0.251 | 0.388 | 0.226 | 0.284 | **0.842** | 0.137 | 0.406 | 0.064 | -0.326 |
| **PIoEC_6** | 0.171 | 0.303 | 0.222 | 0.261 | **0.768** | 0.128 | 0.385 | 0.006 | -0.24 |
| **PIoEC_7** | 0.236 | 0.327 | 0.24 | 0.302 | **0.804** | 0.087 | 0.329 | 0.004 | -0.336 |

**(c)** Fornell-Larcker Criterion

|  | **Affect** | **AtA** | **AtTC** | **BP** | **PIoEC** | **PIoFP** | **PRtTRE** | **PfMCRL** | **RP** |
| --- | --- | --- | --- | --- | --- | --- | --- | --- | --- |
| **Affect** | 1 |  |  |  |  |  |  |  |  |
| **AtA** | 0.533 | 0.914 |  |  |  |  |  |  |  |
| **AtTC** | 0.052 | 0.128 | 0.855 |  |  |  |  |  |  |
| **BP** | 0.476 | 0.778 | 0.099 | 0.889 |  |  |  |  |  |
| **PIoEC** | 0.28 | 0.433 | 0.319 | 0.337 | 0.8 |  |  |  |  |
| **PIoFP** | 0.101 | -0.025 | 0.051 | -0.032 | 0.169 | 0.749 |  |  |  |
| **PRtTRE** | 0.137 | 0.266 | 0.028 | 0.282 | 0.34 | -0.02 | 0.831 |  |  |
| **PfMCRL** | 0.092 | -0.043 | 0.135 | -0.037 | 0.002 | 0.302 | 0.011 | 0.873 |  |
| **RP** | -0.42 | -0.594 | -0.091 | -0.543 | -0.36 | 0.211 | -0.16 | 0.185 | 0.91 |

**(d)** Heterotrait-Monotrait Ratio

|  | **Affect** | **AtA** | **AtTC** | **BP** | **PIoEC** | **PIoFP** | **PRtTRE** | **PfMCRL** | **RP** |
| --- | --- | --- | --- | --- | --- | --- | --- | --- | --- |
| **Affect** |  |  |  |  |  |  |  |  |  |
| **AtA** | 0.561 |  |  |  |  |  |  |  |  |
| **AtTC** | 0.055 | 0.142 |  |  |  |  |  |  |  |
| **BP** | 0.499 | 0.858 | 0.109 |  |  |  |  |  |  |
| **PIoEC** | 0.292 | 0.478 | 0.357 | 0.369 |  |  |  |  |  |
| **PIoFP** | 0.121 | 0.086 | 0.103 | 0.084 | 0.216 |  |  |  |  |
| **PRtTRE** | 0.14 | 0.295 | 0.102 | 0.318 | 0.367 | 0.13 |  |  |  |
| **PfMCRL** | 0.102 | 0.052 | 0.15 | 0.048 | 0.063 | 0.341 | 0.141 |  |  |
| **RP** | 0.434 | 0.648 | 0.101 | 0.586 | 0.391 | 0.228 | 0.152 | 0.208 |  |

**(e)** Inner variance inflation factor (VIF) values

|  | **Affect** | **AtA** | **AtTC** | **BP** | **PIoEC** | **PIoFP** | **PRtTRE** | **PfMCRL** | **RP** |
| --- | --- | --- | --- | --- | --- | --- | --- | --- | --- |
| **Affect** |  | 1.444 |  | 1.101 |  |  |  |  | 1.101 |
| **AtA** |  |  |  |  |  |  |  |  |  |
| **AtTC** | 1.15 | 1.154 |  | 1.153 | 1 | 1 | 1 | 1 | 1.153 |
| **BP** |  | 1.682 |  |  |  |  |  |  |  |
| **PIoEC** | 1.327 | 1.547 |  | 1.41 |  |  |  |  | 1.41 |
| **PIoFP** | 1.149 | 1.25 |  | 1.151 |  |  |  |  | 1.151 |
| **PRtTRE** | 1.151 | 1.198 |  | 1.153 |  |  |  |  | 1.153 |
| **PfMCRL** | 1.132 | 1.173 |  | 1.141 |  |  |  |  | 1.141 |
| **RP** |  | 1.772 |  |  |  |  |  |  |  |

**(f)** Predictive power of the model

|  | **R-square** | **R-square adjusted** |
| --- | --- | --- |
| **Affect** | 0.092 | 0.076 |
| **AtA** | **0.684** | **0.675** |
| **BP** | 0.31 | 0.296 |
| **PIoEC** | 0.102 | 0.099 |
| **PIoFP** | 0.003 | -0.001 |
| **PRtTRE** | 0.001 | -0.003 |
| **PfMCRL** | 0.018 | 0.015 |
| **RP** | 0.345 | 0.332 |

**Table S7.** Results of multigroup analysis between segments

| **Hypotheses** | | **β** | | | |  | **Difference in β** | | |
| --- | --- | --- | --- | --- | --- | --- | --- | --- | --- |
|  |  | **Whole sample** | **S1** | **S2** | **S3** |  | **S1-S2** | **S1-S3** | **S2-S3** |
| **H1a** | **Affect -> BP** | 0.402*** | 0.682*** | 0.606*** | 0.016 |  | 0.076 | 0.667*** | 0.591*** |
| **H1b** | **Affect -> RP** | -0.282*** | -0.732*** | -0.519*** | 0.042 |  | -0.213*** | -0.774*** | -0.561*** |
| **H1c** | **Affect -> AtA** | 0.188*** | 0.200*** | 0.197*** | 0.099*** |  | 0.003 | 0.101 | 0.098* |
| **H1d** | **BP -> AtA** | 0.572*** | 0.388*** | 0.533*** | 0.597*** |  | -0.145* | -0.209** | -0.064 |
| **H1e** | **RP -> AtA** | -0.112*** | -0.251*** | -0.131*** | -0.063* |  | -0.12 | -0.188*** | -0.068 |
| **H2a** | **PfMCRL -> Affect** | 0.017 | -0.101* | 0.031 | 0.055 |  | -0.132 | -0.155* | -0.023 |
| **H2b** | **PfMCRL -> BP** | 0.031 | 0.119*** | -0.021 | 0.044 |  | 0.140** | 0.075 | -0.065 |
| **H2c** | **PfMCRL -> RP** | 0.140*** | -0.037 | 0.110* | 0.189*** |  | -0.147** | -0.226*** | -0.079 |
| **H2d** | **PRtTRE -> Affect** | 0.060* | -0.045 | 0.235*** | 0.032 |  | -0.281*** | -0.077 | 0.203*** |
| **H2e** | **PRtTRE -> BP** | 0.101*** | 0.166*** | 0.194*** | -0.075* |  | -0.028 | 0.240*** | 0.268*** |
| **H2f** | **PRtTRE -> RP** | 0.097*** | -0.087* | -0.061 | 0.370*** |  | -0.026 | -0.457*** | -0.431*** |
| **H3a** | **PfMCRL -> AtA** | -0.02 | 0.011 | -0.087** | 0.009 |  | 0.098* | 0.002 | -0.095* |
| **H3b** | **PRtTRE -> AtA** | 0.036* | -0.017 | 0.111*** | 0.001 |  | -0.128** | -0.018 | 0.109** |
| **H4a** | **PioEC -> Affect** | 0.236*** | 0.392*** | 0.285*** | 0.067 |  | 0.107 | 0.325*** | 0.218** |
| **H4b** | **PioEC -> BP** | 0.298*** | 0.105* | 0.146*** | 0.477*** |  | -0.041 | -0.372*** | -0.331*** |
| **H4c** | **PioEC -> RP** | -0.307*** | -0.051 | -0.160*** | -0.490*** |  | 0.109* | 0.439*** | 0.330*** |
| **H4d** | **PioFP -> Affect** | 0.154*** | 0.002 | 0.021 | 0.451*** |  | -0.019 | -0.449*** | -0.430*** |
| **H4e** | **PioFP -> BP** | 0.031 | 0.075 | -0.027 | 0.250*** |  | 0.102 | -0.176** | -0.277*** |
| **H4f** | **PioFP -> RP** | 0.212*** | 0 | -0.071 | 0.310*** |  | 0.071 | -0.309*** | -0.381*** |
| **H5a** | **PioEC -> AtA** | 0.059** | 0.149*** | 0.023 | 0.049 |  | 0.127* | 0.1 | -0.026 |
| **H5b** | **PioFP -> AtA** | 0.081*** | -0.001 | 0.05 | 0.206*** |  | -0.051 | -0.207*** | -0.156** |
| **H6a** | **AtTC -> PfMCRL** | 0.191*** | 0.232*** | 0.03 | 0.293*** |  | 0.202** | -0.061 | -0.263*** |
| **H6b** | **AtTC -> PRtTRE** | 0.166*** | 0.016 | 0.086 | 0.348*** |  | -0.07 | -0.332*** | -0.262*** |
| **H7a** | **AtTC -> PioEC** | 0.395*** | 0.285*** | 0.153*** | 0.670*** |  | 0.132 | -0.385*** | -0.517*** |
| **H7b** | **AtTC -> PioFP** | 0.150*** | 0.155* | -0.082 | 0.368*** |  | 0.237* | -0.213** | -0.450*** |
| **H8a** | **AtTC -> Affect** | 0.062* | 0.141* | 0.068 | -0.02 |  | 0.073 | 0.160* | 0.088 |
| **H8b** | **AtTC -> BP** | 0.033 | -0.084* | 0.056 | 0.024 |  | -0.141** | -0.108 | 0.032 |
| **H8c** | **AtTC -> RP** | -0.063 ** | 0.028 | -0.197*** | -0.054 |  | 0.225*** | 0.082 | -0.143* |
| **H9** | **AtTC -> AtA** | -0.003 | -0.029 | 0.008 | -0.021 |  | -0.037 | -0.008 | 0.029 |

***Note:*** AtTC refers to attachment to the countryside; PRtTRE refers to perceived risk to the rural environment; PfMCRL refers to preference for maintaining the current rural landscape; PioEC refers to perceived importance of environmental conservation; PioFP refers to perceived importance of food productivity; Affect refers to affect evoked by agroforestry; BP refers to benefit perception of agroforestry; RP refers to risk perception of agroforestry; AtA refers to attitudes towards agroforestry.

**Table S8.** Values of target constructs across segments

| **Constructs** | **S1** | **S2** | **S3** | **Whole sample** |
| --- | --- | --- | --- | --- |
| Attachment to the countryside | 4.30  (0.80) | 4.30  (0.77) | 4.27  (0.68) | 4.29  (0.74) |
| Perceived risk to the rural environment | 3.48  (0.90) | 3.47  (0.90) | 3.54  (0.87) | 3.50  (0.89) |
| Preference for maintaining current rural landscape | 3.51^ab^  (0.85) | 3.48^b^  (0.80) | 3.63^a^  (0.83) | 3.55  (0.83) |
| Perceived importance of environmental conservation | 4.25  (0.57) | 4.18  (0.58) | 4.18  (0.62) | 4.20  (0.59) |
| Perceived importance of food productivity | 3.59^b^  (0.70) | 3.58^b^  (0.68) | 3.72^a^  (0.67) | 3.64  (0.69) |
| Affect evoked by agroforestry | 3.81^a^  (0.97) | 3.65^b^  (0.99) | 3.67^ab^  (1.07) | 3.70  (1.02) |
| Benefit perception of agroforestry | 3.90  (0.78) | 3.79  (0.74) | 3.89  (0.70) | 3.86  (0.74) |
| Risk perception of agroforestry | 2.41^c^  (0.96) | 2.60^b^  (0.87) | 2.83^a^  (0.98) | 2.64  (0.96) |
| Attitudes towards agroforestry | 3.89^ab^  (0.69) | 3.79^b^  (0.66) | 3.90^a^  (0.65) | 3.86  (0.67) |

***Note***: ^a–c^ Values with the same letter as superscript indicate not significantly different means, and different superscripts indicate significantly different means between the segments, following ANOVA Games-Howell post hoc tests at *p* < 0.05. S1 refers to “cautious conservation-oriented citizens”; S2 refers to “citizens sensitive to threats to the rural environment”; S3 refers to “countryside-engaged eco-productive citizens”.

**Table S9.** Proportions of participants belonging to three citizen segments

| **UK Regions** | **No. of Participants** | | |  | **Percentages of Participants** | | |
| --- | --- | --- | --- | --- | --- | --- | --- |
|  | **S1** | **S2** | **S3** |  | **S1** | **S2** | **S3** |
| Northern Ireland | 11 | 12 | 25 |  | 22.9% | 25.0% | 52.1% |
| Scotland | 33 | 39 | 50 |  | 27.0% | 32.0% | 41.0% |
| North East England | 60 | 50 | 92 |  | 29.7% | 24.8% | 45.5% |
| North West England | 46 | 49 | 69 |  | 28.0% | 29.9% | 42.1% |
| Yorkshire and The Humber | 19 | 19 | 28 |  | 28.8% | 28.8% | 42.4% |
| East Midlands | 36 | 44 | 53 |  | 27.1% | 33.1% | 39.8% |
| West Midlands | 29 | 48 | 43 |  | 24.2% | 40.0% | 35.8% |
| Wales | 14 | 26 | 31 |  | 19.7% | 36.6% | 43.7% |
| East of England | 31 | 35 | 47 |  | 27.4% | 31.0% | 41.6% |
| London | 68 | 63 | 71 |  | 33.7% | 31.2% | 35.1% |
| South West England | 35 | 43 | 64 |  | 24.6% | 30.3% | 45.1% |
| South East England | 35 | 41 | 50 |  | 27.8% | 32.5% | 39.7% |

**Table S10. Key results of multinomial logistic regression**

**(a)** Likelihood ratio tests

| **Effect** | **Model Fitting Criteria** | **Likelihood Ratio Tests** | | |
| --- | --- | --- | --- | --- |
|  | **-2 Log Likelihood of Reduced Model** | **Chi-Square** | **df** | **Sig.** |
| Intercept | 1493.293a | 0 | 0 | . |
| The ratio of risk perception to  attachment to the countryside | 2089.928b | 596.635 | 262 | <.001 |
| The ratio of risk perception to preference for  maintaining the current rural landscape | 1965.537b | 472.244 | 216 | <.001 |
| The ratio of risk perception to the perceived  importance of environmental conservation | 2023.512b | 530.219 | 212 | <.001 |
| The ratio of risk perception to the perceived importance of food productivity | 2039.865b | 546.572 | 268 | <.001 |

**Note:** The chi-square statistic is the difference in -2 log-likelihoods between the final model and a reduced model. The reduced model is formed by omitting an effect from the final model. The null hypothesis is that all parameters of that effect are 0.

**a** This reduced model is equivalent to the final model because omitting the effect does not increase the degrees of freedom.

**b** Unexpected singularities in the Hessian matrix are encountered. This indicates that either some predictor variables should be excluded or some categories should be merged.

**(b)** Classification

|  |  | **Predicted** | | | |
| --- | --- | --- | --- | --- | --- |
|  |  | **1** | **2** | **3** | Percent Correct |
| **Observed** | **1** | 280 | 75 | 62 | 67.10% |
|  | **2** | 57 | 329 | 83 | 70.10% |
|  | **3** | 58 | 62 | 503 | 80.70% |
|  | Overall Percentage | 26.20% | 30.90% | 42.90% | **73.70%** |
