# Supplementary material for: Driving climate resilience: citizen attitudes toward agroforestry and their policy implications in the UK
Source: Agron Sustain Dev. 2026 Jul 13;46(4):53. doi: 10.1007/s13593-026-01130-w (PMC13364824; doi:10.1007/s13593-026-01130-w)
Supplement: Supplementary file 2 — (DOCX 28.3 KB) [file 13593_2026_1130_MOESM2_ESM.docx]

**Supplementary File 2**

1. **Determining the number of segments**

To determine the number of segments to retain from the data, FIMIX-PLS was used to obtain fit indices running from one- to five-segment solutions. The results of the modified Akaike information criterion with factor 3 (AIC3) and the consistent Akaike information criterion (CAIC) did not indicate the same number of segments, and neither did AIC3 and the Bayesian information criterion (BIC) (see Table S4). As the minimum description length with factor 5 (MDL5) underestimates, and the Akaike information criterion (AIC) overestimates, the correct number of segments (Matthews et al., 2016), three or four segments could be suitable solutions. Given the lower BIC value and higher normed entropy statistic (EN), as well as considering the parsimony principle to ensure the results remain interpretable and generalisable (Matthews et al., 2016; Sarstedt et al., 2022), three segments were selected as the optimal solution for the data.

1. **Segment-specific effects of constructs**

As FIMIX-PLS often overfits one segment, thereby producing an extremely high R^2^ value, PLS-POS, a hill-climbing approach that gradually reallocates objects between the segments to maximise the solution’s weighted R^2^, was used to run segmentation using a three-segment solution. PLS-POS achieved three segments which accounted for 27.6% (n = 417), 31.1% (n = 469) and 41.3% (n = 623) of the sample. Reliability and validity criteria for the measurement models of each segment were met (Table S5). The resulting models showed no critical collinearity issues between the assessed constructs (VIF < 5) and had a good fit (SRMR = 0.05) (Table S5). The values of R^2^ for three segments were 0.68, 0.73 and 0.62, respectively, resulting in a weighted average R^2^ at 0.67, above the R^2^ (0.65) for the whole-sample SEM. For each segment, direct effects between constructs were obtained. Multigroup analysis was then conducted to examine whether these effects had significant differences across segments. Full details of effects between constructs for each segment can be found in Table S6. Indirect and total effects of constructs on attitudes towards agroforestry were also obtained for comparison among the whole sample and three segments (Table 4).

Concerning the direct effects on attitudes towards agroforestry, benefit perception had the biggest positive impact, followed by risk perception with a negative impact and evoked affect with a positive impact for S1. The results of multi-group analysis (Table S6) showed that the positive effect of benefit perception on participants’ attitudes towards agroforestry in S1 was significantly smaller than those in S2 and S3, while the direct negative effect of risk perception was significantly greater for S1 compared to S3. Participants’ perceived importance of environmental conservation in farming practice had a direct positive effect on attitudes towards agroforestry in S1 and had no significant effect in S2 and S3. Taking indirect effects into account, affect evoked agroforestry indicated the biggest positive impact on attitudes towards agroforestry in S1 (as was for S2), followed by the perceived importance of environmental conservation and benefit perception of agroforestry. As such, S1 was named as “*cautious conservation-oriented citizens*” whose attitudes towards agroforestry were more positively influenced by perceived importance of environmental conservation while also more negatively affected by risk perception of agroforestry.

Concerning the direct effects on attitudes towards agroforestry, benefit perception had the biggest positive impact, followed by evoked affect with a positive impact and risk perception with a negative impact for S2. Perceived risk to the rural environment had positive impacts on affect evoked by, and attitudes towards, agroforestry only in S2. Attachment to the countryside reduced participants’ risk perception of agroforestry. Taking indirect effects into account, affect evoked by agroforestry indicated the biggest positive impact on attitudes towards agroforestry, followed by the benefit perception of agroforestry and perceived risk to the rural environment. The other two segments showed no total effects of perceived risk to the rural environment on attitudes towards agroforestry. Thus, S2 was named as “*citizens sensitive to threats to the rural environment*” where affect and attitudes associated with agroforestry were positively affected by perceived risk to the rural environment, to a significantly greater extent compared S1 and S3.

In S3, benefit perception of agroforestry had the biggest direct positive impact on participants’ attitudes towards agroforestry, followed by the perceived importance of food productivity in farming and affect evoked by agroforestry. Here, evoked affect had a smaller positive impact on attitudes towards agroforestry compared to S1 and S2. The evoked affect had no significant impacts on participants’ benefit and risk perceptions of agroforestry in S3. In contrast, it had a positive impact on benefit perception and a negative impact on risk perception for both S1 and S2. Attachment to the countryside tended to be more influential on participants’ perceptions and preferences related to the countryside and farming in this segment. For instance, the positive influence of attachment to the countryside on their perceived importance of environmental conservation and food productivity and perceived risk to the rural environment for participants in S3 was significantly greater compared to S1 and S2. Taking indirect effects into account, participants’ benefit perception of agroforestry indicated the biggest positive impact on attitudes towards agroforestry, followed by the perceived importance of food productivity, and the perceived importance of environmental conservation and attachment to the countryside. The positive total effect of perceived importance of food productivity on attitudes towards agroforestry was only found in S3, and that of attachment to the countryside in S3 was greater compared to S1 and S2. As such, S3 was named as “*countryside-engaged eco-productive citizens*” whose attitudes towards agroforestry were largely driven by attachment to the countryside, as well as the perceived importance of both environmental conservation and high productivity in farming.

1. **Ex post analysis**

Differences in the values of constructs included in the model and socio-demographic attributes across segments were examined to better characterise the three identified segments (Table S7). Given the unequal sample sizes within each segment, Welsh one-way ANOVA and the Games-Howell post hoc test were used to compare participants’ attachment to the countryside, preference for maintaining the current rural landscape, perceived risk to the rural environment, perceived importance of environmental conservation and food productivity in farming practice, affect evoked by agroforestry, benefit and risk perceptions of, and attitudes towards, agroforestry. The results of Welsh one-way ANOVA indicated significant differences across segments regarding participants’ preference for maintaining the current rural landscape, perceived importance of food productivity, affect, risk perception, and attitudes associated with agroforestry (see Table S7). The results of the Games-Howell post hoc tests indicated that participants in S3 had significantly greater preference for maintaining the current rural landscape compared to those in S2 and placed greater importance on food productivity in farming than the other two segments. Agroforestry evoked significantly more positive affect among participants in S1 compared to S2. Participants in S3 had the highest risk perception of agroforestry, followed by S2 and S1, and held significantly more positive attitudes than those in S2. In addition, the results of Chi-square showed that participants across segments had no significant differences regarding the gender distribution or whether they were frequent countryside visitors. The results of the Welsh one-way ANOVA and the Games-Howell post hoc test indicated a significant difference across segments regarding participants’ age (*M* = 49 for S1 and S2 and *M* = 46 for S3), with the average age for S3 significantly younger than S2. The results of a multinomial logistic regression indicated that participants who received higher education were more likely to belong to S3 compared to S2. The proportions of participants belonging to the three citizen segments varied across the 12 UK regions (Table S8). The highest percentages were observed in London for S1 (33.7%), the West Midlands for S2 (40.0%), and Northern Ireland for S3 (52.1%). The lowest percentages were found in Wales for S1 (19.7%), the Northeast of England for S2 (24.8%), and London for S3 (35.1%).

There is potential to partition the data using a combination of several variables to create groupings that largely correspond to the latent segments obtained through PLS-POS. An overlap of 60% between the PLS-POS partition and the one produced by the explanatory variables is considered satisfactory (Matthews et al., 2016; Sarstedt et al., 2022). Based on a multinomial logistic regression, four variables (the ratio of risk perception to attachment to the countryside, the ratio of risk perception to preference for maintaining the current rural landscape, the ratio of risk perception to the perceived importance of environmental conservation in farming, and the ratio of risk perception to the perceived importance of food productivity) successfully predicted 67.1%, 70.1% and 80.7% of the cases for S1, S2 and S3, respectively. Detailed analysis can be found in Table S9.
